# Predictors of Adherence Among Patients With Multiple Sclerosis Using the BETACONNECT^®^ Autoinjector: A Prospective Observational Cohort Study

**DOI:** 10.3389/fneur.2021.643126

**Published:** 2021-02-24

**Authors:** Wolfgang Köhler, Kirsten Bayer-Gersmann, Thomas Neußer, Markus Schürks, Tjalf Ziemssen

**Affiliations:** ^1^Department of Neurology, University of Leipzig Medical Center, Leipzig, Germany; ^2^Institut Dr. Schauerte, Munich, Germany; ^3^Bayer Vital GmbH, Leverkusen, Germany; ^4^Multiple Sclerosis Center Dresden, Center of Clinical Neuroscience, Neurological Clinic, University Hospital Carl Gustav Carus, Dresden University of Technology, Dresden, Germany

**Keywords:** multiple sclerosis, disease modifying drugs, interferon beta-1b, autoinjector, BETACONNECT®, adherence, persistence, compliance

## Abstract

**Background:** In patients with multiple sclerosis (MS), non-adherence to disease-modifying drug therapy is associated with an increased rate of MS relapses. Early identification of patients at risk of non-adherence would allow provision of timely and individualized support. The aim of the BETAPREDICT study was to investigate potential predictors of adherence in patients with MS in Germany treated with interferon β-1b (IFNβ-1b) using the BETACONNECT® autoinjector.

**Methods:** BETAPREDICT was a national, multi-center, prospective, non-interventional, single-arm, 24-month cohort study of patients with relapsing–remitting MS or clinically isolated syndrome receiving IFNβ-1b via the BETACONNECT® autoinjector (ClinicalTrials.gov: NCT02486640). Injection data were captured by the autoinjector. The primary objective was to determine baseline predictors of compliance, persistence, and adherence to IFNβ-1b treatment after 12- and 24 months using multivariable-adjusted regression. Secondary objectives included evaluation of satisfaction with the autoinjector, injection site pain, vitamin and nutrient supplementation, clinical course, and patient-related outcome measures.

**Results:** Of 165 patients enrolled, 153 were available for analysis (120 with autoinjector data). Seventy-two patients left the study prematurely. Compliance (*N* = 120), persistence (*N* = 153), and adherence (*N* = 120) at 24 months were 89.1, 53.6, and 41.7%, respectively. Compliance at 12- and 24 months was predicted by intake of vitamin D supplements and absence of specific injection site reactions. Positive predictors of persistence included age (at 12- and 24 months) and previous duration of treatment (at 12 months), while intake of vitamins/nutrients other than vitamin D was a negative predictor (at 12 months). Positive predictors of adherence at 24 months were age and being experienced with IFNβ-1b. Higher scores in specific SF-36 subscales were positive predictors of medication-taking behavior at 24 months. Satisfaction with the autoinjector was high at baseline and 24 months (median score: 9 out of 10).

**Conclusions:** Compliance with IFNβ-1b treatment among participants still under observation remained high over a 24-month period, while persistence and adherence continuously declined. Multiple factors affected medication-taking behavior, including patient characteristics, treatment history, injection site reactions, patients' perception of their health and support programs. The importance of these factors may differ among patients according to their individual situation.

## Introduction

Multiple sclerosis (MS) is an autoimmune inflammatory demyelinating and degenerative disorder of the central nervous system, which typically starts in young adulthood (1).

There is no cure for MS, but disease-modifying drugs (DMDs), including the well-established injectable therapies interferon beta (IFNβ) and glatiramer acetate (GA), can delay disease progression if administered with strict adherence to the prescribed dose and administration schedule (2, 3). However, because of the chronic nature of the disease, patients with MS often become non-adherent to DMD therapy over time. For example, non-adherence to IFNβ or GA therapy after 2 years was reported for 60–70% of patients in an analysis of German pharmacy claims data (4). Similarly, in a large US claims database 42% of patients initiating IFNβ or GA were non-adherent, based on medication possession ratio over the 1–3-year study period (5). In the Global Adherence Project, 25% of patients were non-adherent to their prescribed IFNβ or GA treatment regimen over a 4-week period (6).

Patients who miss doses or interrupt DMD therapy have a higher rate of MS relapses than patients who adhere to their treatment regimen (7, 8). Furthermore, adherence has been shown to have a direct effect on healthcare resource utilization and may thus affect healthcare costs (3, 7, 8). There is therefore a continuing need to increase adherence to DMD therapies in MS. Strategies to enhance adherence to DMD therapy include patient education and support as well as improving treatment tolerability and convenience, for example by adjusting drug formulation and/or using injection devices (9).

Use of autoinjectors was identified as a predictor of adherence to IFNβ-1b in a prospective, multinational, 2-year cohort study (10). The BETACONNECT® is an electronic autoinjector for the subcutaneous administration of IFNβ-1b. It allows individualized adjustment of injection settings, automatically records each injection, and has an electronic reminder function (11). In the recent BETAEVAL 24-week observational study, the majority of patients continued using the BETACONNECT® autoinjector for IFNβ-1b treatment during the study period and showed a high level of adherence (12). Early identification of patients at risk of non-adherence would allow provision of timely and individualized support.

The aim of the BETAPREDICT study was to gain a more comprehensive understanding of potential predictors of adherence by investigating, over a 24-month period, a representative cohort of patients in Germany who had MS treated with IFNβ-1b and were using the BETACONNECT® autoinjector.

## Methods

### Study Design and Participants

BETAPREDICT was designed as a national, multi-center, prospective, non-interventional, observational, single-arm, 24-month cohort study (ClinicalTrials.gov Identifier: NCT02486640). The study was conducted in German MS centers and private neurological offices/clinics specialized in the treatment of patients with MS. It was planned to include 250 participants. Patients were enrolled consecutively to avoid selection bias. To decrease reporting bias, a selection of the documented data from 150 patients from 21 sites (out of a total of 26 active sites that enrolled patients for the study) were reviewed for completeness, plausibility and adherence to the study protocol, and were verified against source documents.

Prior to the start of the study, documented approval was obtained from the independent ethics committee at the Technische Universität Dresden (approval number: EK183042015). All enrolled patients provided written informed consent.

### Eligibility

Patients aged ≥18 years with relapsing–remitting MS (RRMS) or clinically isolated syndrome (CIS) who were initiating or receiving ongoing treatment with IFNβ-1b as part of their routine care and who were willing to use the BETACONNECT® autoinjector were eligible for inclusion in the study. Patients were excluded if they were receiving any DMD other than IFNβ-1b, if they had any contraindications for IFNβ-1b, or if they were participating in any other clinical or non-interventional study evaluating MS therapy. The decision to treat with IFNβ-1b was made at the discretion of the attending physician, according to his/her medical practice.

The full analysis set included all patients who injected IFNβ-1b at least once, provided informed consent before the initial visit, and had any subsequent follow-up information in the electronic case report forms (eCRF). The safety analysis set included all patients who injected IFNβ-1b at least once during the observation period, with that injection recorded in the eCRF or in the BETACONNECT® log.

### Objectives and Endpoints

The primary objective was to determine baseline predictors of medication-taking behavior by evaluating compliance, persistence and adherence to IFNβ-1b treatment via the BETACONNECT® autoinjector after 12- and 24 months. Analysis of persistence was performed for the full analysis set; compliance and adherence were assessed only for patients with BETACONNECT® data.

Compliance and persistence were defined as recommended by the International Society for Pharmacoeconomics and Outcomes Research Medication Compliance and Persistence Work Group (13). Compliance refers to the extent to which treatment recommendations are followed, with respect to timing, dosage and frequency. Persistence refers to the act of continuing the treatment for the prescribed duration (13). The term adherence is used in the present study to describe patients who were both compliant and persistent, as in our previous study (12) and aligning with definitions based on the medication possession ratio (4, 5). Compliance, persistence, and adherence were calculated from baseline to each study visit.

The primary endpoint was compliance, expressed as a percentage. For each period, compliance was calculated as the number of injections performed (captured by the BETACONNECT® autoinjector) divided by the number of injections expected multiplied by 100. To characterize non-compliance further, the number of missed doses was also calculated.

Co-primary endpoints were persistence of therapy (yes/no) and adherence to therapy (yes/no). Non-persistence was defined as premature discontinuation of therapy or exceeding the permissible gap between doses (measured as cessation of therapy for ≥4 weeks). Adherence to therapy was defined as injection of ≥80% of expected doses and continuation in the study with persistence of therapy at the time of evaluation.

In some cases persistence could not be determined with certainty due to lack of reliable data, and was classed as “possible.” For the final calculation of persistence and adherence, a conservative approach was used, with patients in the “possible” category assumed to be non-persistent.

Secondary objectives were to evaluate at each visit: satisfaction with the BETACONNECT® autoinjector (scale of 0 to 10, where “0” means “not satisfied at all” and “10” means “entirely satisfied”); injection site pain (scale of 0 to 10, where “0” means “no pain at all” and “10” means the “worst possible pain”); analgesic use prior to IFNβ-1b application; flu-like symptoms following IFNβ-1b application; intake of vitamin D, other vitamins, and nutrients; and whether adherence to IFNβ-1b treatment was associated with depression [assessed using the Allgemeine Depressionsskala, which is the validated German translation of the Center for Epidemiologic Studies Depression Scale (CES-D) (14)], health-related quality of life [assessed using the 36-item short form health survey (SF-36) (15)], self-efficacy mechanisms [assessed using the MS Self-Efficacy Scale (16)], fatigue [assessed using the Würzburg Fatigue Inventory for MS (WEIMuS) (17)], cognition [assessed using the Symbol Digit Modalities Test (SDMT) (18); completed at initial visit, 12 months, and final visit only], or utility values [assessed using the 5-dimension EuroQol questionnaire (EQ-5D-5L) (19) using the German value set (20); completed at initial and final visits only].

### Data Collection

For each patient, the investigator documented data in standardized electronic case report forms (eCRF) at the initial, follow-up and final visits. Documented follow-up visits occurred ~6 months apart during routine practice. The final documented visit occurred ~24 months after the initial visit, at discontinuation of therapy, or at the end of the study (whichever was earliest).

The investigator collected historic data (demographic and clinical characteristics) from medical records if available, or else by interviewing the patient. Likewise, the investigator collected treatment-related data during the initial visit and follow-up visits. Injection-related data stored in the BETACONNECT® autoinjector were downloaded from the device during scheduled visits and were then uploaded into the eCRF. At each visit, patients were asked to complete a questionnaire including items on satisfaction, use of analgesics prior to injection, injection site pain and flu-like symptoms, as well as the CES-D, SF-36, MS Self-Efficacy Scale, WEIMuS, SDMT, and EQ-5D-5L instruments. Adverse events were documented at follow-up visits and the final visit.

### Statistical Analysis

Statistical analyses were exploratory and descriptive. Statistical analyses were performed in SAS 9.4 (SAS Institute Inc., Cary, NC, USA). Analyses of statistical significance were conducted only for the association of baseline covariates with medication-taking behavior; thresholds for statistical significance are described below.

First, one-way analysis of variance (ANOVA; categorical variables) and linear regression (continuous variables) were used to investigate univariate associations of baseline covariates (as independent variables) with compliance (as the dependent variable) at 12- and 24 months. Second, all covariates that showed a univariate association with *p* ≤ 0.1 were added to a regression model employing a stepwise selection procedure (entry level: *p* = 0.25; stay level: *p* = 0.1) to determine predictors of compliance. Predictors of the co-primary endpoints persistence and adherence were identified by univariate and multivariable-adjusted logistic regression models, similar to the approach used for compliance.

The linear regression analyses described above were performed in the subgroup of patients with data from BETACONNECT®. The following covariates were considered in the regression models: age (linear), sex (female or male), body mass index (BMI), marital status (married/partnership or single), employment status (employed, retired, keeping house, student, seeking work, self-employed, other, or not reported), educational level (no certificate, elementary education, secondary education, university entrance qualification, college or university education, or not reported), concomitant diseases (yes or no), number of concomitant diseases (0, 1, or ≥2; or continuous), baseline expanded disability status scale (EDSS) score (≥3 or <3), number of relapses during year prior to enrolment (0, 1, or >1), diagnosis (RRMS or CIS), intake of vitamin D supplements (yes or no; dosage), intake of other nutrients or vitamins (yes or no), smoking (never, past, or present), IFNβ-1b treatment status (treatment-naïve or already receiving treatment), concomitant medication (yes or no), number of concomitant medications (0, 1, or ≥2), time since MS diagnosis (months), duration of treatment from first injection until last injection or final visit (days), previous usage of the BETACONNECT® autoinjector (yes or no), usage of electronic features of the BETACONNECT® autoinjector, injection site pain (numerical analog scale), local skin reactions (yes or no; if yes: redness, other discoloration, hematoma, induration, lipodystrophy, or necrosis; if yes: mild, moderate, or severe), flu-like symptoms (yes or no), participation in the Patient Support and Disease Management Program (PSDMP) BETAPLUS® (yes or no), SDMT score at initial visit, SF-36 subscale scores, CES-D score, MS Self-Efficacy Scale score, and WEIMuS subscale and total scores.

As for the analysis of persistence and adherence, for modeling purposes patients in the “possible” persistence category were assumed to be non-persistent.

Secondary endpoints were evaluated descriptively at each visit. Subgroup analyses were conducted to investigate secondary endpoints according to patients' age, sex, experience with the BETACONNECT® autoinjector, participation in the PSDMP BETAPLUS®, and according to their persistence and their adherence at 12- and 24 months.

## Results

### Patient Disposition

**Figure 1:**
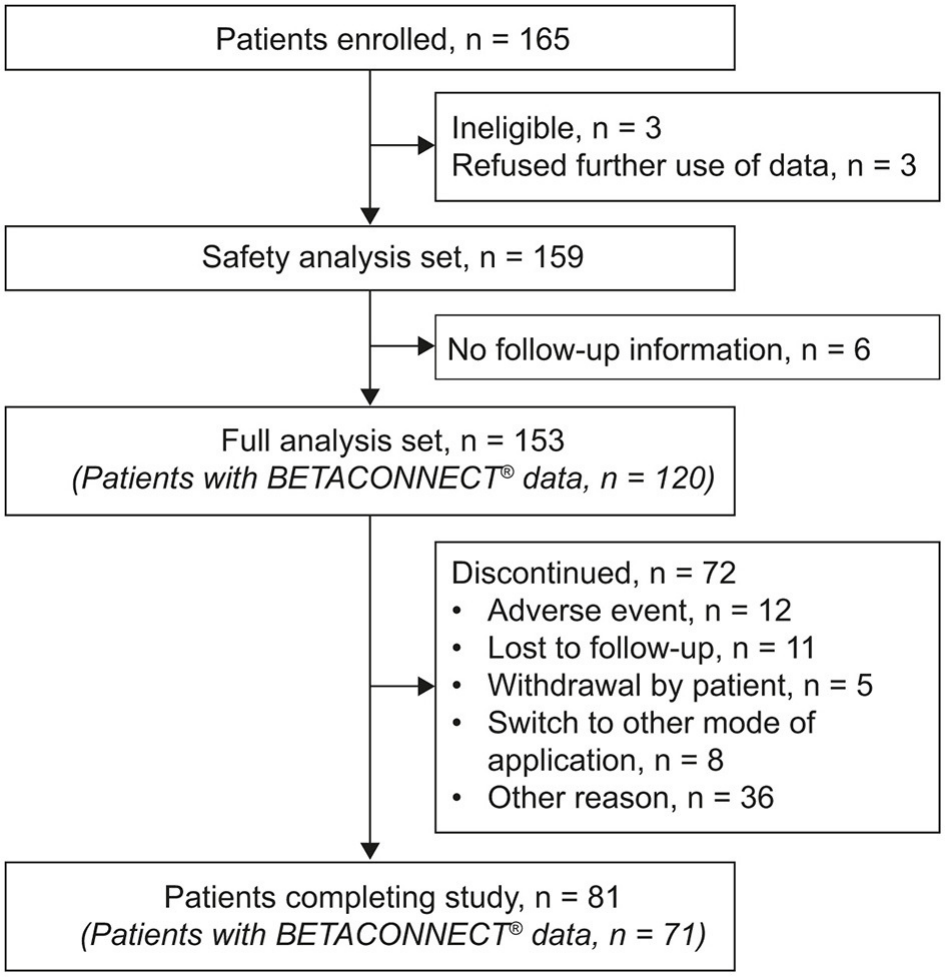
Flow chart describing patient disposition in the BETAPREDICT study.

In total, 165 patients were enrolled in the BETAPREDICT study. Of those, 12 patients were excluded because of refusal of further use of data (three patients), ineligibility (three patients) or absence of any follow-up information (six patients), resulting in 153 patients included in the full analysis (Figure 1). A total of 81 patients completed the observation period, with a mean (standard deviation [SD]) follow-up duration of 24.7 (2.9) months; 72 patients left the study prematurely, with a mean (SD) follow-up duration of 12.3 (6.8) months. The corresponding median (interquartile range [IQR]) durations of follow-up were 24.1 (23.5–24.8) and 12.0 (6.2–17.7) months.

### Baseline Demographics and Characteristics

The demographic and clinical characteristics of the patient population at the time of enrollment are summarized in Table 1. The mean age of participants was 42.5 years, and the majority (*n* = 102; 66.7%) were female. Most patients (*n* = 145; 94.8%) had RRMS, with the remainder having CIS. The overall mean time since the first clinical event suggestive of MS was 95.5 months. The majority of patients (*n* = 114; 74.5%) were already injecting IFNβ-1b; of these, 94.7% had previously used an autoinjector, most commonly BETACONNECT® (83.3%). Participants in the PSDMP BETAPLUS® (*n* = 73; duration of treatment: mean 52.5 months, median 22.8 months) had been treated with IFNβ-1b longer than those not participating in the PSDMP BETAPLUS® (*n* = 72; duration of treatment: mean 35.7 months, median 17.3 months).

**Table 1:** Baseline demographics and clinical characteristics of participants of the BETAPREDICT study.

**Characteristic**	**BETAPREDICT study population (*N* = 153)**
**Age, years**	
Mean (SD)	42.5 (11.8)
Median (range)	42 (20–67)
**Sex**, ***n*** **(%)**	
Female	102 (66.7%)
Male	51 (33.3%)
**BMI, kg/m**^**2**^	
Mean (SD)	26.6 (6.0)
Median (range)	25.6 (18.1–53.2)
**Diagnosis**, ***n*** **(%)**	
RRMS	145 (94.8%)
CIS	8 (5.2%)
**Time since first clinical event suggestive of MS, months**^**a**^	
Mean (SD)	95.5 (98.9)
Median (range)	60.4 (0.2–456.9)
**EDSS, median (range)**	1.5 (0.0–6.5)
**Already injecting IFNβ-1b**, ***n*** **(%)**	114 (74.5%)
**Most recently used autoinjector for IFNβ-1b treatment among patients who received IFNβ-1b previously**, ***n*** **(%)**	
Any	108 (94.7%)
BETACONNECT^®^	95 (83.3%)
BETACOMFORT^®^	6 (5.3%)
BETAJECT Comfort^®^	4 (3.5%)
BETAJECT lite^®^	1 (0.9%)
Other	2 (1.8%)
**Participation in PSDMP BETAPLUS**^®^, ***n*** **(%)**	73 (47.7%)
**Employment status**, ***n*** **(%)**^**a**^	
Employed	93 (60.8%)
Retired	23 (15.0%)
Keeping house	9 (5.9%)
Student	5 (3.3%)
Seeking work	3 (2.0%)
Self-employed	7 (4.6%)
Other	2 (1.3%)

a *N = 151*.

*Numbers (%) may not add up to total numbers (100%) due to missing values. BMI, body mass index; CIS, clinically isolated syndrome; EDSS, expanded disability status scale; IFN, interferon; MS, multiple sclerosis; PSDMP, Patient Support and Disease Management Program; RRMS, relapsing remitting multiple sclerosis; SD, standard deviation*.

### Compliance, Persistence, and Adherence

**Figure 2:**
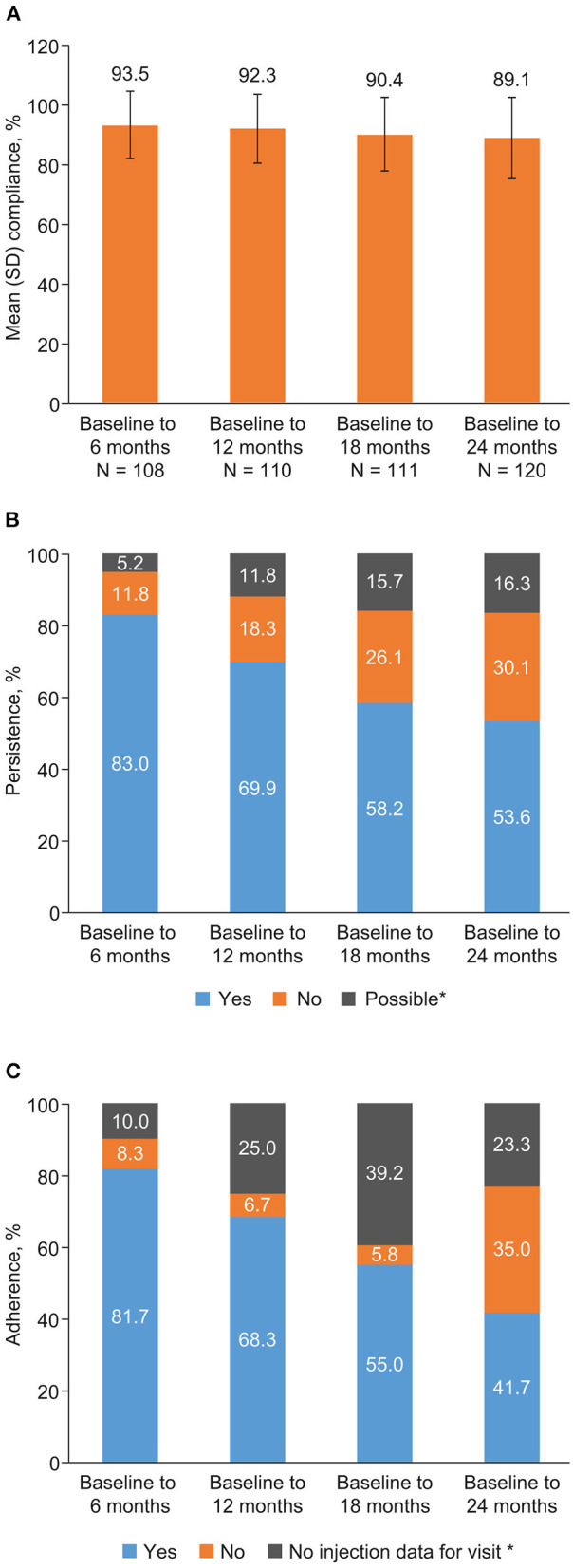
**(A)** Compliance, **(B)** persistence (*N* = 153), and **(C)** adherence (*N* = 120) during the BETAPREDICT study. Compliance: *N* indicates number of patients with available data for each time period. Persistence: “possible” indicates a lack of reliable data, making it impossible to determine persistence with certainty. Adherence: for some patient visits, no injection data were assigned to the given follow-up interval, either because no injections were recorded for the relevant time interval or because the visit was missed.

Among the 120 patients with data available from the BETACONNECT® autoinjector, mean (SD) compliance from baseline to 6-, 12-, and 18 months, and to the final visit (24 months), was 93.5% (11.7%), 92.3% (11.7%), 90.4% (12.7%), and 89.1% (14.0%), respectively (Figure 2A). The mean (SD) number of missed doses from baseline to 6-, 12-, and 18 months, and to the final visit (24 months), was 6.1 (12.1), 12.4 (19.1), 22.3 (31.7), and 30.2 (39.8), respectively.

The proportion of persistent patients from baseline to 6-, 12-, and 18 months, and to the final visit (24 months), was 83.0, 69.9, 58.2, and 53.6%, respectively (Figure 2B). Due to a lack of reliable data, 5.2, 11.8, 15.7, and 16.3% of patients over the four time intervals were only possibly persistent, and were considered as non-persistent for analysis purposes.

Adherence was seen for 81.7, 68.3, 55.0, and 41.7% of patients, respectively, from baseline to 6-, 12-, and 18 months, and to the final visit (Figure 2C). Supplementary Table 1 provides a stratified analysis of compliance, persistence, and adherence for specific subgroups of patients—results were generally similar across subgroups.

### Predictors of Compliance, Persistence, and Adherence

#### Compliance

Results of univariate analyses are shown in the Supplementary Material. The results of the final multivariable models after stepwise selection are shown in Table 2.

**Table 2:** Predictors of compliance, persistence, and adherence in the final multivariable models after stepwise selection procedure in patients using the BETACONNECT® autoinjector.

**Predictor**	**Compliance**		**Persistence**		**Adherence**	
	**12 months**	**24 months**	**12 months**	**24 months**	**12 months**	**24 months**
Age (years)	—	—	+0.039 (0.020)	+0.058 (0.019)	—	+0.049 (0.020)
Duration of treatment (days)	—	—	+0.0005 (0.0002)	—	—	—
Intake of vitamin D supplements (effect of “yes”)	+9.716 (3.658)	+5.764 (2.571)	—	—	—	—
Intake of other nutrients or vitamins (effect of “yes”)	—	—	−1.827 (0.558)	—	—	—
Injection site hematoma (effect of “yes”)	−14.549 (5.644)	−13.294 (5.679)	—	—	—	—
Injection site lipodystrophy (effect of “yes”)	−20.300 (9.289)	−27.346 (9.615)	—	—	—	—
SF-36: general health perceptions at initial visit (standardized scale: 0–100)	—	−0.209 (0.082)	—	—	—	—
SF-36: limitations in usual role activities because of emotional problems at initial visit (standardized scale: 0–100)	—	—	—	+0.034 (0.012)	—	+0.027 (0.013)
New patients vs. patients already on IFNβ-1b (effect of “already injecting IFNβ-1b”)	—	—	—	+1.209 (0.499)	—	+1.389 (0.659)
Usage of electronic features of BETACONNECT^®^ (effect of “yes”)	—	—	—	—	−1.211 (0.628)	—
Participation in the PSDMP BETAPLUS^®^ (effect of “yes”)	—	—	—	—	+0.985 (0.470)	—

*Data are presented as effect estimates (standard error)*.

*PSDMP, Patient Support and Disease Management Program; SF-36, 36-item short form health survey*.

Use of vitamin D supplements and the absence of hematoma and lipodystrophy at the injection site were associated with compliance at 12- and 24 months in univariate analysis (Supplementary Tables 3–6). In addition, having moderate intensity local skin reactions was associated with decreased compliance at 12 months, while a larger number of concomitant diseases and lower scores in the SF-36 general health and usual role limitations subscales were associated with increased compliance at 24 months. After stepwise selection, use of vitamin D supplements and the absence of hematoma and lipodystrophy at the injection site were retained as predictors in both final models; in addition, having lower scores in the SF-36 general health subscale was retained as a predictor in the 24-month model.

#### Persistence

Univariate analysis found that persistence at 12- and 24 months was associated with having fewer relapses in the year prior to enrollment, and with older age, longer time since diagnosis and longer duration of treatment (Supplementary Tables 7–12). In addition, 12-month persistence was associated with not using nutrients or vitamins other than vitamin D and with the absence of necrosis at the injection site. Twenty four-month persistence was additionally associated with baseline EDSS scores ≥3.0, with patients previously using IFNβ-1b, with patients being experienced with the BETACONNECT® autoinjector, and with higher scores in the SF-36 usual role limitations subscale. After stepwise selection, age was retained as a predictor in both the 12- and 24-month models [12 months: odds ratio (OR) 1.04, 95% confidence interval (CI) 1.00–1.08; 24 months: OR 1.06, 95% CI 1.02–1.10]. Further, use of nutrients or vitamins other than vitamin D (OR 0.16, 95% CI 0.05–0.48) and duration of treatment (OR 1.00, 95% CI 1.00–1.00) were retained as negative and positive predictors, respectively, in the 12-month model, and previous usage of IFNβ-1b (OR 3.35, 95% CI 1.26–8.91) and higher scores in the SF-36 usual role limitations subscale (OR 1.035, 95% CI 1.01–1.06) were retained as positive predictors in the 24-month model.

#### Adherence

In the univariate analysis, female sex, not using the BETACONNECT® electronic features, participation in the PSDMP BETAPLUS® and a higher number of concomitant diseases were associated with greater adherence at 12 months (Supplementary Tables 13–15). After stepwise selection, using the BETACONNECT® electronic features (OR 0.30, 95% CI 0.09–1.02) was a negative predictor, while participation in the PSDMP BETAPLUS® (OR 2.68, 95% CI 1.07–6.73) was a positive predictor in the 12-month analysis.

In the 24-month univariate analysis of adherence, previous usage of IFNβ-1b and the BETACONNECT®, not using the BETACONNECT® electronic features, age and higher scores in the SF-36 usual role limitations subscale were positively associated with adherence (Supplementary Tables 16–18). After stepwise selection, age (OR 1.05, 95% CI 1.01–1.09), previous usage of IFNβ-1b (OR 4.01, 95% CI 1.10–14.60) and having higher scores in the SF-36 usual role limitations subscale (OR 1.03, 95% CI 1.00–1.05) were identified as positive predictors of adherence.

### Satisfaction With the BETACONNECT® Autoinjector

Satisfaction with the BETACONNECT® autoinjector was generally high, with median (IQR) satisfaction scores (scored out of 10) of 9.0 (8.0–10.0) at baseline and 9.0 (7.5–10.0) at the final visit. Median satisfaction scores were generally similar across subgroups (data not shown).

### Injection Site Pain and Use of Analgesics

Mean (SD) patient-reported injection site pain (scored out of 10) was 3.5 (2.4) at baseline and 3.9 (2.5) at the final visit. Injection site pain scores were generally similar across subgroups (data not shown).

At the initial visit, 18/131 patients (13.7%) reported using analgesics prior to injection; the corresponding proportions at 12 months and at the final visit were 13.0% (12/92) and 8.0% (7/88), respectively. Use of prophylactic analgesics appeared to be more common in female patients than in male participants (18.4 vs. 4.5% at the initial visit; no statistical testing was conducted).

### Vitamin and Nutrient Supplementation

At the initial visit, 35/153 patients (22.9%) were using vitamin D supplementation; the corresponding proportions at 12- and 24 months were 36/108 (33.3%) and 23/81 (28.4%), respectively. In total, 19/153 (12.4%), 7/108 (6.5%), and 6/81 (7.4%) patients were using other vitamin or nutrient supplementation at baseline, 12- and 24 months, respectively. The most common supplement was vitamin B12 (nine patients at baseline).

### Clinical Course

At the initial visit, the median EDSS score was 1.5 (IQR, 1.0–2.5; data from 121 patients); at the final visit (24 months) the median score was 2.0 (IQR, 1.3–2.5; *n* = 64 patients). A higher proportion of patients with a baseline EDSS score of ≥ 3.0 (*n* = 24) were adherent at 24 months, compared with those with baseline EDSS scores of 0.0–2.5 (*n* = 96; 63 vs. 46%); no difference was seen in adherence at 12 months (71 vs. 69%).

From the initial visit to 12 months and from 12- to 24 months, patients had a mean (SD) of 0.2 (0.6) and 0.1 (0.3) relapses, respectively. A total of 25 patients had relapses during the observation period, with 13 patients having a single relapse and 12 having multiple relapses.

### Patient-Related Outcome Measures

Patient-related outcome measures (CES-D, SF-36, MS Self-Efficacy Scale, WEIMuS, SDMT, and EQ-5D-5L) are summarized in Supplementary Table 2. Patient-related outcome measure results were generally similar across subgroups and in patients who were and were not adherent (data not shown).

### Adverse Events

In the safety analysis set (*n* = 159), serious drug-related treatment-emergent adverse events (TEAEs) were reported in two patients; non-serious drug-related TEAEs were reported in 60 patients. The most common drug-related TEAE was injection site erythema (28 patients). The serious drug-related TEAEs were injection site necrosis and MS relapse. Overall, the reported drug-related TEAEs were consistent with the known safety profile of IFNβ-1b; no new safety signals were identified.

## Discussion

The BETAPREDICT study investigated medication-taking behavior and its predictors in patients with MS receiving IFNβ-1b treatment using the BETACONNECT® autoinjector over a follow-up period of 24 months. Persistence was high at 6 months (83.0%), and 53.6% of patients remained persistent up to the final visit (24 months). Mean compliance among participants still under observation remained high throughout the study (89.1% from baseline to the final visit). Adherence—the proportion of patients who were both persistent and at least 80% compliant—was high during the first 6 months of the study (81.7%) and decreased in line with persistence, with 41.7% of patients adherent over the full 24-month observation period.

The BETAPREDICT study showed higher compliance with IFNβ-1b therapy after 24 months (89.1%) than a recent study using the RebiSmart® device. The RebiSmart® study was conducted in a Mexican cohort. In this study adherence was defined similar to compliance in our study; they found a mean adherence of 79.5% after a median follow-up of 27.5 months (21). Compliance in both of these recent studies, which used electronic injection data, was higher than in previous retrospective observational studies based on medication possession ratio. For example, one study in Germany reported a median medication possession ratio of 0.64 (4) and one from the USA a mean medication possession ratio of 0.70 (5). The previous study in Germany reported 33.4% persistence with IFNβ-1b therapy (4), compared with 53.6% in our study. However, direct comparison of results between studies is difficult, and the differences may be explained by the study designs, definition of outcome variables, and a time trend (for example). The previous studies (4, 5) were retrospective analyses of claims databases using the medication possession ratio as a surrogate for compliance. This approach harbors measurement uncertainty, whereas injection behavior in our study was automatically and prospectively recorded by the BETACONNECT®, which may be more accurate. In addition, the prospective design of the BETAPREDICT study may have contributed; the patients were aware that they were participating in a study, which may have influenced their medication-taking behavior. A further explanation may be that the usage of the BETACONNECT® autoinjector eases the injections for patients as shown previously (11).

A previous prospective study reported a comparatively high rate of adherence (61.8%; defined as completion of study protocol and medication) over 24 months (10), compared with 41.7% in our study. One potential explanation for this difference is our conservative approach of classifying patients with inconsistent data in their case report forms as non-adherent, which has likely underestimated the true adherence. The earlier study measured adherence without assessing the proportion of expected doses injected (10); this definition of adherence is closer to the endpoint of persistence in the current study (53.6% of patients were persistent at 24 months). In addition, at the time of the previous study, which was published in 2011, few alternative disease-modifying therapies were available; this may potentially mean that patients in that cohort were less likely to switch therapies, compared with the current study.

The current study has identified age as a predictor of persistence at 12- and 24 months. These findings are consistent with several previous studies that reported older age as a predictor of medication-taking behavior. While the BETAEVAL study (12) reported this for persistence among patients treated with IFNβ-1b using the BETACONNECT® autoinjector at 24 weeks, further studies have shown a positive association of age with persistence and adherence for up to 2.5 years (5, 10, 22).

The BETAEVAL study (12) found that being newly treated with IFNβ-1b was a predictor of adherence over a 6-month period. By contrast, we found that previous use of IFNβ-1b (i.e., being IFNβ-1b experienced) predicted persistence and adherence at 24 months. This may be explained by the fact that medication-taking behavior in newly prescribed patients often declines within the first year (4), suggesting retention of an increasing proportion of experienced patients. As might be expected, hematoma and lipodystrophy injection site-reactions were strong negative predictors of compliance in the current study.

The finding that participation in the PSDMP BETAPLUS® positively predicts adherence appears conceptually to support the idea of patient support programs aiming to help patients stay on their medication for improved treatment outcomes.

We found that higher SF-36 scores for general health perception and limitations in usual role activities because of emotional problems positively predicted medication-taking behavior at 24 months. This appears intuitive, considering that people who feel better find it easier to activate their coping resources and be self-efficacious (23). Being adherent would be a result of this.

Intake of vitamin D supplements was a strong positive predictor of compliance. A possible explanation is that there may be a correlation between compliance and vitamin D use, because patients who are more health conscious may pay close attention both to regular injections and additional activities believed to be health-promoting. Health conscious patients may be aware of the results of previous studies conducted among patients with MS treated with IFNβ-1b: vitamin D levels have been found to be inversely correlated with MS activity observed on magnetic resonance imaging (24), while low vitamin D levels early in the disease course are a strong risk factor for long-term MS activity and disease progression (25). The negative association between use of other supplements and persistence at 12 months is surprising, although it is possible that the use of other supplements reflects generally poor health, consistent with the results for the SF-36 general health subscale.

Satisfaction with the BETACONNECT® autoinjector and overall health-related quality of life assessed using the EQ-5D-5L were high at baseline and remained high at the final visit. Treatment satisfaction has previously been found to be associated with health-related quality of life in patients with MS (26), and increased treatment satisfaction and effectiveness have been shown to be associated with a longer duration of medication use (27).

### Strengths

First, our study used minimal eligibility criteria in order to be as representative as possible, and therefore included patients from a more diversified and less selected patient population than would be typical in a clinical trial setting. The prerequisite to use the BETACONNECT® may be considered to impact the representativeness of our study population. However, the impact is likely to be small since about 80% of patients treated with IFNβ-1b in Germany use the autoinjector. Second, our study population appears similar to previously published cohorts with MS. For example, fatigue (measured by the WEIMUS), cognition (measured by the SDMT), and depression (measured by the CES-D) among participants of our study are similar to measurements in previously described populations treated with IFNβ-1b (12, 28). In addition, participants' self-rated health-related quality of life (measured by the EQ-5D VAS) in our study was very similar to that of a sample from the German population (20). Third, assessments of compliance, persistence and adherence were based on direct measurement of actual injections and physicians' reports rather than indirect proxy measures, such as the medication possession ratio. Fourth, injection-related data were automatically recorded by the BETACONNECT® autoinjector, eliminating the effect of recall bias, a common problem in observational studies.

### Limitations

First, the selection of sites and departments specialized in the management and treatment of MS may have limited the representativeness of the study population. However, patients with MS in Germany are typically treated in such centers. Second, it is possible that the study population includes a high proportion of patients with low disease activity, given the mean time since the first clinical event suggestive of MS was ~8 years; this would also be consistent with the low relapse rate observed in the study. Third, patients with prior usage of the BETACONNECT® autoinjector may have been positively selected with respect to adverse events (i.e., they may still have been using the device because they experienced no or only mild adverse effects). Fourth, we were only able to enroll 165 of the planned 250 patients. However, recruitment was slower than expected, which reflects the real-world treatment and prescribing situation. We decided not to extend the recruitment period further. Finally, our study did not include a control group. However, the main aim of the study was not to compare users of the BETACONNECT® to non-users, but to describe prospectively adherence and predictors of adherence among patients using IFNβ-1b over a 24-month period.

### Conclusions

The results of the BETAPREDICT study suggest that age, longer duration of treatment and having previously used IFNβ-1b predict continuation of the treatment (i.e., persistence). Regular administration of injections according to treatment recommendations (i.e., compliance) appears to be negatively determined by occurrence of local skin reactions at the injection site and positively determined by intake of vitamin D supplements. This pattern can be seen after 12- and 24 months. Adherence, which shares aspects of persistence and compliance, appears to be positively predicted by participation in a dedicated PSDMP and negatively predicted by usage of electronic features of the BETACONNECT® during the first 12 months. In addition, previous usage of IFNβ-1b therapy seems to predict adherence over 24 months (as seen for persistence). Better perceptions of specific aspects of health-related quality of life (SF-36 subscale scores) appear to be positive predictors of medication-taking behavior at 24 months. Psychoeducational programs may be helpful to promote recognition of the relationship between long-term adherence and beneficial treatment effects.

## Data Availability Statement

The original contributions presented in the study are included in the article/Supplementary Material, further inquiries can be directed to the corresponding author/s.

## Ethics Statement

The studies involving human participants were reviewed and approved by Independent ethics committee at the Technische Universität Dresden, Germany. The patients/participants provided their written informed consent to participate in this study.

## Author Contributions

WK, MS, and TZ were responsible for the conception and design of the study. TN and MS were responsible for the study coordination and conduct. KB-G and MS were responsible for the data analysis. All authors interpreted the data, contributed to and critically reviewed the manuscript during its development, and approved the final version of the manuscript for submission. BETAPREDICT Study Group members provided the patient data.

## Betapredict Study Group Members

Samir Al-Boutros, Kathlen Bach, Sven Ehrlich, Birte Elias-Hamp, Ralph Erbacher, Johannes Fischer, Frank Halbgewachs, Werner E. Hofmann, Judith Jeske, Boris-A. Kallmann, Ulrich Kausch, Michael Kirsch, Andreas Kowalik, Gabriele Luber, Mike Matzke, Thomas Müller, Patrick Oschmann, Cornelia Rau, Frank Schmitz, Karl-Otto Sigel, Joachim Springub, Erik Strauß, Ansgar Thümen, Klaus Tiel-Wilck, Roland Wenzelburger, and Uwe Zettl.

## Conflict of Interest

WK received speaker honoraria and grant support from Bayer Vital, Grifols, Merck Serono, Novartis, Roche, and Teva. KB-G was an employee at Institut Dr. Schauerte during the analysis phase of the study. TZ received personal compensation from Almirall, Biogen, Bayer, Celgene, Novartis, Roche, Sanofi, and Teva for consulting and speaking services. TZ received additional financial support for research activities from Biogen, Novartis, Roche, Teva, and Sanofi. TN and MS are full-time employees of Bayer Vital GmbH. The authors declare that this study received funding from Bayer Vital GmbH. The funder was involved in the following way: conception, design, coordination and conduct of the study, analysis and interpretation of the data, drafting of the manuscript.
