# Supplementary material for: Predictors of Adherence Among Patients With Multiple Sclerosis Using the BETACONNECT® Autoinjector: A Prospective Observational Cohort Study
Source: Front Neurol. 2021 Feb 24;12:643126. doi: 10.3389/fneur.2021.643126 (PMC7943887; doi:10.3389/fneur.2021.643126)
Supplement: Supplementary file 1 [file Table_1.docx]

Supplementary Tables

# Additional study results

**Supplementary Table 1.** Compliance, persistence, and adherence stratified by age, sex, previous experience with the BETACONNECT^®^, and participation in the PSDMP BETAPLUS^®^

|  | **Mean (SD) compliance, % *[n with data available]*** | | **Mean (SD) time from initial visit to last injection, months *[n with data available]*** | **Adherence, n (%) *[n with data available]*** | |
| --- | --- | --- | --- | --- | --- |
|  | **From baseline to 12‑month visit** | **From baseline to final visit** |  | **From baseline to 12‑month visit** | **From baseline to final visit** |
| Total | 92.3 (11.7) *[110]* | 89.1 (14.0) *[120]* | 10.6 (6.3) *[46]* | 82 (91.1%) *[90]* | 50 (54.3%) *[92]* |
| Age, years |  |  |  |  |  |
| < 40 | 93.3 (11.2) *[45]* | 89.1 (15.6) *[54]* | 10.2 (6.0) *[27]* | 32 (88.9%) *[36]* | 19 (45.2%) *[42]* |
| ≥ 40 | 91.6 (12.1) *[65]* | 89.2 (12.7) *[66]* | 11.3 (6.7) *[19]* | 50 (92.6%) *[54]* | 31 (62%) *[50]* |
| Sex |  |  |  |  |  |
| Female | 92.6 (12.1) *[76]* | 89.0 (15.4) *[81]* | 10.7 (6.8) *[31]* | 60 (92.3%) *[65]* | 33 (54.1%) *[61]* |
| Male | 91.6 (10.9) *[34]* | 89.4 (10.8) *[39]* | 10.4 (5.1) *[15]* | 22 (88%) *[25]* | 17 (54.8%) *[31]* |
| Previous experience with the BETACONNECT^®^ autoinjector |  |  |  |  |  |
| Yes | 91.6 (12.3) *[71]* | 88.7 (13.0) *[74]* | 11.0 (6.6) *[28]* | 53 (93.0%) *[57]* | 34 (63.0%) *[54]* |
| No | 93.5 (10.5) *[39]* | 89.8 (15.7) *[46]* | 10.0 (5.8) *[18]* | 29 (87.9%) *[33]* | 16 (42.1%) *[38]* |
| Participation in the PSDMP BETAPLUS^®^ |  |  |  |  |  |
| Yes | 92.1 (13.0) *[64]* | 89.7 (13.4) *[65]* | 12.0 (6.8) *[21]* | 48 (92.3%) *[52]* | 28 (60.9%) *[46]* |
| No | 92.6 (9.7) *[46]* | 88.5 (14.8) *[55]* | 9.4 (5.6) *[25]* | 34 (89.5%) *[38]* | 22 (47.8%) *[46]* |

PSDMP, Patient Support and Disease Management Program; SD, standard deviation.

**Supplementary Table 2.** Patient-related outcome measures

|  | **Initial visit** | **6-month visit** | **12-month visit** | **18-month visit** | **Final visit** |
| --- | --- | --- | --- | --- | --- |
| CES-D (scale: 0–‍60) | 14.5 (9.0) *[142]* | 13.8 (9.7) *[120]* | 13.7 (10.5) *[93]* | 13.4 (10.0) *[78]* | 12.7 (10.1) *[90]* |
| SF-36 (scale: 0–‍100) |  |  |  |  |  |
| Physical functioning | 81.6 (26.9) *[139]* | 80.0 (27.5) *[116]* | 80.7 (27.8) *[94]* | 79.3 (28.8) *[78]* | 82.2 (25.5) *[91]* |
| Role-physical | 37.1 (19.1) *[139]* | 38.7 (18.8) *[116]* | 37.9 (19.9) *[93]* | 36.6 (20.6) *[78]* | 35.6 (20.9) *[90]* |
| Bodily pain | 64.2 (20.6) *[125]* | 63.5 (21.0) *[109]* | 64.9 (21.9) *[84]* | 62.8 (22.1) *[71]* | 64.0 (22.1) *[85]* |
| General health | 47.8 (16.2) *[138]* | 48.1 (17.5) [*113]* | 48.6 (16.4) *[92]* | 48.8 (18.6) *[77]* | 49.6 (18.5) *[89]* |
| Vitality | 45.4 (17.8) *[140]* | 45.6 (17.9) *[114]* | 48.3 (17.5) *[92]* | 47.7 (18.1) *[76]* | 47.4 (18.4) *[91]* |
| Social functioning | 39.3 (7.8) *[139]* | 37.8 (7.6) *[116]* | 38.7 (10.3) *[94]* | 36.4 (7.0) *[78]* | 37.8 (10.0) *[91]* |
| Role-emotional | 40.3 (18.1) *[138]* | 39.4 (19.0) *[116]* | 38.0 (19.9) *[93]* | 40.3 (18.8) *[77]* | 39.3 (20.2) *[90]* |
| Mental health | 58.9 (16.3) *[140]* | 58.0 (16.4) *[114]* | 59.9 (16.4) *[92]* | 59.2 (16.5) *[76]* | 60.1 (17.0) *[91]* |
| MS Self-Efficacy Scale, total score (scale: 14–‍84) | 61.7 (11.5) *[145]* | 61.4 (11.4) *[117]* | 62.1 (11.2) *[94]* | 61.2 (11.7) *[76]* | 62.3 (11.5) *[89]* |
| WEIMuS (scale: 0–‍68) | 13.0 (2.0–33.5) *[144]* | 13.0 (3.0–31.0) *[118]* | 12.5 (2.0–34.0) *[94]* | 11.0 (1.0–36.0) *[77]* | 13.0 (2.0–39.0) *[91]* |
| SDMT total (scale: 0–‍110) | 49.1 (12.3) *[113]* | – | 50.9 (12.5) *[58]* | – | 51.1 (10.0) *[49]* |
| EQ-5D-5L weighted index (scale: 0–‍1) | 0.9 (0.2) *[139]* | – | – | – | 0.9 (0.2) *[82]* |
| EQ-5D VAS (scale: 0–‍100) | 78.3 (16.9) *[144]* | – | – | – | 77.1 (18.1) *[83]* |

Data are presented as mean (standard deviation) or median (interquartile range) *[n with data available]*.

CES-D, Center for Epidemiologic Studies Depression Scale; EQ-5D-5L, 5-dimension, 5-level EuroQol questionnaire; MS, multiple sclerosis; SDMT, Symbol Digit Modalities Test; SF-36, 36-item short form health survey; WEIMuS, Würzburg Fatigue Inventory for Multiple Sclerosis.

# Detailed modelling results – compliance at 12 months

Supplementary Table 3. One way ANOVA for compliance at 12 months (categorical independent variables)

| **Variable** | **Label** | **N** | **DF** | **Anova SS** | **Mean Square** | **F-Statistics** | **Pr > F** |
| --- | --- | --- | --- | --- | --- | --- | --- |
| SEX | Sex (male, female) | 119 | 1 | 20.740885 | 20.740885 | 0.10 | 0.7465 |
| MARITAL0 | Marital status (married/partnership, single) | 114 | 1 | 19.763605 | 19.763605 | 0.11 | 0.7443 |
| EMPLOY0 | Employment status (employed, retired, keeping house, student, seeking work, self-employed, other, not reported) | 114 | 7 | 381.238853 | 54.462693 | 0.28 | 0.9591 |
| EDUCAT | Educational level (elementary education, secondary education, college or university education, not reported) | 113 | 4 | 91.225120 | 22.806280 | 0.12 | 0.9753 |
| MEDHISTny | Concomitant diseases (no, yes) | 119 | 1 | 383.712585 | 383.712585 | 1.97 | 0.1628 |
| MEDHISTNumberga | Number of concomitant diseases (0,1,>=2) | 119 | 2 | 390.752381 | 195.376190 | 1.00 | 0.3724 |
| EDSS0ga | Baseline EDSS (0-2.5,>=3) | 119 | 1 | 5.920281 | 5.920281 | 0.03 | 0.8629 |
| EVENTNO0ga | Number of relapses during last two years prior to enrolment (missing/unknown,0,1,>1) | 119 | 3 | 630.226188 | 210.075396 | 1.07 | 0.3633 |
| MSStatus | CIS vs. RRMS | 119 | 1 | 63.788640 | 63.788640 | 0.32 | 0.5706 |
| VITDny | Intake of vitamin D supplements at any visit (no, yes) | 119 | 1 | 871.305209 | 871.305209 | 4.58 | 0.0345 |
| VITOTHny | Intake of other nutrients or vitamins (no, yes) | 119 | 1 | 17.070345 | 17.070345 | 0.09 | 0.7693 |
| SMOKING | Smoking (never, past, present) | 113 | 2 | 540.869863 | 270.434931 | 1.48 | 0.2329 |
| BETATREAT | New patients vs. patients already on Betaferon® | 119 | 1 | 1.897381 | 1.897381 | 0.01 | 0.9221 |
| ConcMedny | Concomitant medication (no, yes) | 119 | 1 | 26.887400 | 26.887400 | 0.14 | 0.7128 |
| CONCMEDnumberga | Number of concomitant medications (0,1,>=2) | 119 | 2 | 75.014176 | 37.507088 | 0.19 | 0.8283 |
| BetacExpny | Previous usage of BETACONNECT™ (BETACONNECT-naïve, BETACONNECT-experienced) | 119 | 1 | 30.859996 | 30.859996 | 0.16 | 0.6933 |
| PQ5 | Usage of electronic features of BETACONNECT (no, yes) | 98 | 1 | 272.127374 | 272.127374 | 1.34 | 0.2491 |
| SKINREACTny | Injection-site reactions (no, yes) | 119 | 1 | 91.552293 | 91.552293 | 0.46 | 0.4967 |
| REACT1ny | Injection-site reactions (redness; no,yes) | 119 | 1 | 182.085205 | 182.085205 | 0.93 | 0.3373 |
| REACT2ny | Injection-site reactions (other discoloration; no,yes) | 119 | 1 | 72.994405 | 72.994405 | 0.37 | 0.5440 |
| REACT3ny | Injection-site reactions (hematoma; no,yes) | 119 | 1 | 821.486915 | 821.486915 | 4.31 | 0.0401 |
| REACT4ny | Injection-site reactions (induration; no,yes) | 119 | 1 | 59.912260 | 59.912260 | 0.30 | 0.5826 |
| REACT5ny | Injection-site reactions (lipodystrophy; no,yes) | 119 | 1 | 716.891900 | 716.891900 | 3.74 | 0.0555 |
| REACT6ny | Injection-site reactions (necrosis; no,yes) | 119 | 1 | 56.680485 | 56.680485 | 0.29 | 0.5930 |
| INTENSMax | Worst intensity of local skin reactions at any visit (light, moderate, severe) | 50 | 1 | 729.548575 | 729.548575 | 3.68 | 0.0610 |
| PQ3 | Flu-like symptoms (no, yes) | 96 | 1 | 145.954848 | 145.954848 | 0.85 | 0.3589 |
| BETAPLUSny | Participation in the Patient Support and Disease Management Program BETAPLUS® (no, yes) | 119 | 1 | 5.333150 | 5.333150 | 0.03 | 0.8698 |

The numbers of observations used (N) may vary from the total number of patients in the FAS, since patients with missing values for either the dependent and/or independent variables were not imputed and left out of the respective analysis.

Supplementary Table 4. Univariate linear regression for compliance at 12 months (continuous independent variables)

| **Variable** | **Label** | **N** | **DF** | **Effect estimate** | **Standarderror** | **t-Value** | **Pr > \|t\|** |
| --- | --- | --- | --- | --- | --- | --- | --- |
| age | Age (years) | 119 | 1 | 0.02735 | 0.10961 | 0.25 | 0.8034 |
| bmi | BMI (kg/m²) | 110 | 1 | -0.05219 | 0.24728 | -0.21 | 0.8332 |
| MEDHISTNumber | number of concomitant diseases | 119 | 1 | 1.24810 | 0.79943 | 1.56 | 0.1212 |
| VITDDOSE0 | daily dosage of vitamin D intake at initial visit | 29 | 1 | -0.00000769 | 0.00004880 | -0.16 | 0.8760 |
| timesince_diagnosis | Time since diagnosis (months) | 113 | 1 | -0.02360 | 0.01559 | -1.51 | 0.1330 |
| Treatdur_CRF | Duration of treatment (days) | 118 | 1 | -0.00075886 | 0.00074410 | -1.02 | 0.3099 |
| PQ4 | Injection site pain at initial visit (0:“No pain at all“ - 10: „Extremely severe pain) | 98 | 1 | 0.16086 | 0.59098 | 0.27 | 0.7861 |
| SDMTNO0 | SDMT score at initial visit (0-110 correct answers) | 86 | 1 | -0.16246 | 0.13070 | -1.24 | 0.2173 |
| sf36_3 | SF-36: limitations in physical activities because of health problems at initial visit (Standardized scale: 0-100) | 112 | 1 | 0.02792 | 0.04954 | 0.56 | 0.5742 |
| sf36_4 | SF-36: limitations in usual role activities because of physical health problems at initial visit (Standardized scale: 0-100) | 112 | 1 | -0.00641 | 0.06933 | -0.09 | 0.9265 |
| sf36_78 | SF-36: bodily pain at initial visit (Standardized scale: 0-100) | 102 | 1 | -0.06144 | 0.07351 | -0.84 | 0.4053 |
| sf36_11 | SF-36: general health perceptions at initial visit (Standardized scale: 0-100) | 111 | 1 | -0.06661 | 0.08677 | -0.77 | 0.4443 |
| sf36_9vit | SF-36: vitality at initial visit (Standardized scale: 0-100) | 113 | 1 | -0.05023 | 0.07460 | -0.67 | 0.5022 |
| sf36_610 | SF-36: limitations in social activities because of physical or emotional problems at initial visit (Standardized scale: 0-100) | 112 | 1 | -0.11238 | 0.18082 | -0.62 | 0.5356 |
| sf36_5 | SF-36: limitations in usual role activities because of emotional problems at initial visit (Standardized scale: 0-100) | 111 | 1 | -0.10401 | 0.07475 | -1.39 | 0.1669 |
| sf36_9psy | SF-36: general mental health at initial visit (Standardized scale: 0-100) | 113 | 1 | -0.10960 | 0.08054 | -1.36 | 0.1763 |
| cesd_sum | Depression (ADS-L) at initial visit (0:“No depressive symptoms“ - 60:“Maximum amount of depressive symptoms“) | 114 | 1 | 0.19149 | 0.14785 | 1.30 | 0.1979 |
| weimus_ges | Fatigue at initial visit (total sum score from 0 to 68, with higher scores indicating higher degrees of fatigue) | 117 | 1 | -0.00854 | 0.07867 | -0.11 | 0.9138 |
| weimus_kog | Cognitive fatigue at initial visit (subscore from 0 to 36) | 116 | 1 | 0.08122 | 0.15183 | 0.53 | 0.5937 |
| weimus_koer | Physical fatigue at initial visit (subscore from 0 to 32) | 116 | 1 | -0.10967 | 0.14921 | -0.74 | 0.4638 |
| msss | MSSS at initial visit (score from 14 to 84) | 117 | 1 | -0.01923 | 0.11332 | -0.17 | 0.8655 |

# Detailed modelling results – compliance at 24 months

Supplementary Table 5. One way ANOVA for compliance at 24 months (categorical independent variables)

| **Variable** | **N** | **DF** | **Anova SS** | **Mean Square** | **F-Statistics** | **Pr > F** |
| --- | --- | --- | --- | --- | --- | --- |
| SEX | 120 | 1 | 4.996151 | 4.996151 | 0.03 | 0.8741 |
| MARITAL0 | 115 | 1 | 57.726361 | 57.726361 | 0.30 | 0.5880 |
| EMPLOY0 | 115 | 7 | 575.945684 | 82.277955 | 0.41 | 0.8956 |
| EDUCAT | 114 | 4 | 318.985805 | 79.746451 | 0.40 | 0.8094 |
| MEDHISTny | 120 | 1 | 312.406473 | 312.406473 | 1.60 | 0.2088 |
| MEDHISTNumberga | 120 | 2 | 397.813486 | 198.906743 | 1.01 | 0.3667 |
| EDSS0ga | 120 | 1 | 104.513255 | 104.513255 | 0.53 | 0.4683 |
| EVENTNO0ga | 120 | 3 | 341.318420 | 113.772807 | 0.57 | 0.6343 |
| MSStatus | 120 | 1 | 39.890306 | 39.890306 | 0.20 | 0.6543 |
| VITDny | 120 | 1 | 597.808768 | 597.808768 | 3.09 | 0.0812 |
| VITOTHny | 120 | 1 | 177.074409 | 177.074409 | 0.90 | 0.3447 |
| SMOKING | 114 | 2 | 324.546805 | 162.273402 | 0.83 | 0.4399 |
| BETATREAT | 120 | 1 | 12.855081 | 12.855081 | 0.06 | 0.7994 |
| ConcMedny | 120 | 1 | 5.398735 | 5.398735 | 0.03 | 0.8692 |
| CONCMEDnumberga | 120 | 2 | 494.749980 | 247.374990 | 1.26 | 0.2864 |
| BetacExpny | 120 | 1 | 34.195650 | 34.195650 | 0.17 | 0.6785 |
| PQ5 | 98 | 1 | 335.645364 | 335.645364 | 1.71 | 0.1944 |
| SKINREACTny | 120 | 1 | 35.863352 | 35.863352 | 0.18 | 0.6712 |
| REACT1ny | 120 | 1 | 39.315241 | 39.315241 | 0.20 | 0.6566 |
| REACT2ny | 120 | 1 | 158.124074 | 158.124074 | 0.80 | 0.3720 |
| REACT3ny | 120 | 1 | 664.880417 | 664.880417 | 3.45 | 0.0657 |
| REACT4ny | 120 | 1 | 76.377623 | 76.377623 | 0.39 | 0.5354 |
| REACT5ny | 120 | 1 | 1197.973463 | 1197.973463 | 6.37 | 0.0129 |
| REACT6ny | 120 | 1 | 98.861327 | 98.861327 | 0.50 | 0.4806 |
| INTENSMax | 51 | 1 | 312.844115 | 312.844115 | 1.50 | 0.2258 |
| PQ3 | 97 | 1 | 231.051707 | 231.051707 | 1.39 | 0.2419 |
| BETAPLUSny | 120 | 1 | 39.216710 | 39.216710 | 0.20 | 0.6571 |

Supplementary Table 6. Univariate linear regression for compliance at 24 months (continuous independent variables)

| **Variable** | **Label** | **N** | **DF** | **Effect estimate** | **Standarderror** | **t-Value** | **Pr > \|t\|** |
| --- | --- | --- | --- | --- | --- | --- | --- |
| age | Age (years) | 120 | 1 | 0.06012 | 0.10885 | 0.55 | 0.5817 |
| bmi | BMI (kg/m²) | 111 | 1 | 0.08071 | 0.25190 | 0.32 | 0.7493 |
| MEDHISTNumber | number of concomitant diseases | 120 | 1 | 1.57805 | 0.79392 | 1.99 | 0.0492 |
| VITDDOSE0 | daily dosage of vitamin D intake at initial visit | 29 | 1 | 0.00000111 | 0.00005602 | 0.02 | 0.9843 |
| timesince_diagnosis | Time since diagnosis (months) | 114 | 1 | -0.01064 | 0.01553 | -0.68 | 0.4950 |
| Treatdur_CRF | Duration of treatment (days) | 119 | 1 | -0.00045800 | 0.00074617 | -0.61 | 0.5405 |
| PQ4 | Injection site pain at initial visit (0:“No pain at all“ - 10: „Extremely severe pain) | 99 | 1 | 0.09659 | 0.56990 | 0.17 | 0.8658 |
| SDMTNO0 | SDMT score at initial visit (0-110 correct answers) | 87 | 1 | -0.17022 | 0.13320 | -1.28 | 0.2047 |
| sf36_3 | SF-36: limitations in physical activities because of health problems at initial visit (Standardized scale: 0-100) | 113 | 1 | 0.00734 | 0.04939 | 0.15 | 0.8821 |
| sf36_4 | SF-36: limitations in usual role activities because of physical health problems at initial visit (Standardized scale: 0-100) | 113 | 1 | -0.00340 | 0.06896 | -0.05 | 0.9608 |
| sf36_78 | SF-36: bodily pain at initial visit (Standardized scale: 0-100) | 103 | 1 | -0.07226 | 0.07253 | -1.00 | 0.3215 |
| sf36_11 | SF-36: general health perceptions at initial visit (Standardized scale: 0-100) | 112 | 1 | -0.16886 | 0.08508 | -1.98 | 0.0497 |
| sf36_9vit | SF-36: vitality at initial visit (Standardized scale: 0-100) | 114 | 1 | -0.05031 | 0.07420 | -0.68 | 0.4991 |
| sf36_610 | SF-36: limitations in social activities because of physical or emotional problems at initial visit (Standardized scale: 0-100) | 113 | 1 | -0.11235 | 0.16813 | -0.67 | 0.5054 |
| sf36_5 | SF-36: limitations in usual role activities because of emotional problems at initial visit (Standardized scale: 0-100) | 112 | 1 | -0.14763 | 0.07308 | -2.02 | 0.0458 |
| sf36_9psy | SF-36: general mental health at initial visit (Standardized scale: 0-100) | 114 | 1 | -0.12702 | 0.08003 | -1.59 | 0.1153 |
| cesd_sum | Depression (ADS-L) at initial visit (0:“No depressive symptoms“ - 60:“Maximum amount of depressive symptoms“) | 115 | 1 | 0.20951 | 0.14869 | 1.41 | 0.1616 |
| weimus_ges | Fatigue at initial visit (total sum score from 0 to 68, with higher scores indicating higher degrees of fatigue) | 118 | 1 | 0.01752 | 0.07866 | 0.22 | 0.8241 |
| weimus_kog | Cognitive fatigue at initial visit (subscore from 0 to 36) | 117 | 1 | 0.12812 | 0.15140 | 0.85 | 0.3991 |
| weimus_koer | Physical fatigue at initial visit (subscore from 0 to 32) | 117 | 1 | -0.06080 | 0.14940 | -0.41 | 0.6848 |
| msss | MSSS at initial visit (score from 14 to 84) | 118 | 1 | -0.03831 | 0.11199 | -0.34 | 0.7329 |

The numbers of observations used (N) may vary from the total number of patients in the FAS, since patients with missing values for either the dependent and/or independent variables were not imputed and left out of the respective analysis.

# Detailed modelling results – persistence at 12 months

Supplementary Table 7. Logistic regression for persistence at 12 months (categorical independent variables)

| **Effect** | **Label** | **Reference** | **N** | **DF** | **Waldsches ChiSq** | **Pr > ChiSq** |
| --- | --- | --- | --- | --- | --- | --- |
| SEX | Sex (male, female) | 'Male' | 153 | 1 | 0.9902 | 0.3197 |
| MARITAL0 | Marital status (married/partnership, single) | 'Single' | 146 | 1 | 0.0487 | 0.8253 |
| EMPLOY0 | Employment status (employed, retired, keeping house, student, seeking work, self-employed, other, not reported) | 'Employed' | 145 | 7 | 3.8911 | 0.7922 |
| EDUCAT | Educational level (no certificate, elementary education, secondary education, university entrance qualification, college or university education, not reported) | 'Elementary education' | 142 | 5 | 1.2094 | 0.9440 |
| MEDHISTny | Concomitant diseases (no, yes) | 'No' | 153 | 1 | 1.9263 | 0.1652 |
| MEDHISTNumberga | Number of concomitant diseases (0,1,>=2) | '0' | 153 | 2 | 2.0395 | 0.3607 |
| EDSS0ga | Baseline EDSS (0-2.5,>=3) | '0 - 2.5' | 153 | 1 | 1.7555 | 0.1852 |
| EVENTNO0ga | Number of relapses during last two years prior to enrolment (missing/unknown,0,1,>1) | '0' | 153 | 3 | 7.3462 | 0.0616 |
| MSStatus | CIS vs. RRMS | 'CIS' | 153 | 1 | 0.2200 | 0.6390 |
| VITDny | Intake of vitamin D supplements at any visit (no, yes) | 'No' | 153 | 1 | 2.4590 | 0.1169 |
| VITOTHny | Intake of other nutrients or vitamins (no, yes) | 'No' | 153 | 1 | 5.6232 | 0.0177 |
| SMOKING | Smoking (never, past, present) | 'Never' | 142 | 2 | 0.8491 | 0.6541 |
| BETATREAT | New patients vs. patients already on Betaferon® | 'Betaferon-treatment-naïve' | 152 | 1 | 2.0123 | 0.1560 |
| ConcMedny | Concomitant medication (no, yes) | 'No' | 153 | 1 | 0.9096 | 0.3402 |
| CONCMEDnumberga | Number of concomitant medications (0,1,>=2) | '0' | 153 | 2 | 1.2496 | 0.5354 |
| BetacExpny | Previous usage of BETACONNECT™ (BETACONNECT-naïve, BETACONNECT-experienced) | 'BETACONNECT-naïve ' | 153 | 1 | 0.3218 | 0.5705 |
| PQ5 | Usage of electronic features of BETACONNECT (no, yes) | 'No' | 123 | 1 | 1.0967 | 0.2950 |
| SKINREACTny | Injection-site reactions (no, yes) | 'No' | 153 | 1 | 0.0149 | 0.9028 |
| REACT1ny | Injection-site reactions (redness; no,yes) | 'No' | 153 | 1 | 0.0154 | 0.9011 |
| REACT2ny | Injection-site reactions (other discoloration; no,yes) | 'No' | 153 | 1 | 0.0002 | 0.9887 |
| REACT3ny | Injection-site reactions (hematoma; no,yes) | 'No' | 153 | 1 | 0.9126 | 0.3394 |
| REACT4ny | Injection-site reactions (induration; no,yes) | 'No' | 153 | 1 | 0.0258 | 0.8723 |
| REACT5ny | Injection-site reactions (lipodystrophy; no,yes) | 'No' | 153 | 1 | 0.3612 | 0.5479 |
| REACT6ny | Injection-site reactions (necrosis; no,yes) | 'No' | 153 | 1 | 2.9307 | 0.0869 |
| INTENSMax | Worst intensity of local skin reactions at any visit (light, moderate, severe) | 'Light' | 61 | 2 | 1.2249 | 0.5420 |
| PQ3 | Flu-like symptoms (no, yes) | 'No' | 119 | 1 | 0.0716 | 0.7891 |
| BETAPLUSny | Participation in the Patient Support and Disease Management Program BETAPLUS® (no, yes) | 'No' | 153 | 1 | 0.6890 | 0.4065 |

The numbers of observations used (N) may vary from the total number of patients in the FAS, since patients with missing values for either the dependent and/or independent variables were not imputed and left out of the respective analysis.

Supplementary Table 8. Logistic regression for persistence at 12 months (categorical independent variables) – effect estimates

| **Effect** | **Odds ratio estimate** | **LowerCL** | **UpperCL** |
| --- | --- | --- | --- |
| SEX Female vs Male | 1.442 | 0.701 | 2.963 |
| MARITAL0 Married / with a partner vs Single | 1.097 | 0.481 | 2.500 |
| EMPLOY0 Keeping House vs Employed | 0.485 | 0.121 | 1.949 |
| EMPLOY0 Not reported vs Employed | 0.776 | 0.067 | 8.929 |
| EMPLOY0 Other vs Employed | <0.001 | <0.001 | >999.999 |
| EMPLOY0 Retired vs Employed | 1.100 | 0.391 | 3.095 |
| EMPLOY0 Seeking work vs Employed | 0.776 | 0.067 | 8.929 |
| EMPLOY0 Self-employed vs Employed | 2.328 | 0.267 | 20.289 |
| EMPLOY0 Student vs Employed | 0.259 | 0.041 | 1.638 |
| EDUCAT College or university education vs Elementary education | 0.857 | 0.243 | 3.029 |
| EDUCAT General certificate for secondary education vs Elementary education | 0.660 | 0.231 | 1.885 |
| EDUCAT No certificate vs Elementary education | <0.001 | <0.001 | >999.999 |
| EDUCAT Not reported vs Elementary education | 0.526 | 0.096 | 2.882 |
| EDUCAT University entrance qualification vs Elementary education | 1.053 | 0.216 | 5.128 |
| MEDHISTny Yes vs No | 1.645 | 0.814 | 3.323 |
| MEDHISTNumberga 1 vs 0 | 1.884 | 0.681 | 5.208 |
| MEDHISTNumberga >1 vs 0 | 1.526 | 0.684 | 3.404 |
| EDSS0ga 3 - 10 vs 0 - 2.5 | 1.928 | 0.730 | 5.090 |
| EVENTNO0ga 1 vs 0 | 0.563 | 0.228 | 1.386 |
| EVENTNO0ga >1 vs 0 | 0.217 | 0.071 | 0.660 |
| EVENTNO0ga Missing vs 0 | 0.650 | 0.249 | 1.696 |
| MSStatus RRMS vs CIS | 1.423 | 0.326 | 6.222 |
| VITDny Yes vs No | 1.772 | 0.867 | 3.625 |
| VITOTHny Yes vs No | 0.351 | 0.148 | 0.834 |
| SMOKING Current vs Never | 0.824 | 0.340 | 1.994 |
| SMOKING Former vs Never | 1.342 | 0.544 | 3.308 |
| BETATREAT already injecting Betaferon vs Betaferon-treatment-naïve | 1.746 | 0.808 | 3.773 |
| ConcMedny Yes vs No | 1.435 | 0.683 | 3.011 |
| CONCMEDnumberga 1 vs 0 | 1.165 | 0.428 | 3.169 |
| CONCMEDnumberga >1 vs 0 | 1.550 | 0.705 | 3.408 |
| BetacExpny Yes vs No | 1.227 | 0.605 | 2.487 |
| PQ5 Yes vs No | 0.551 | 0.180 | 1.682 |
| SKINREACTny Yes vs No | 1.045 | 0.515 | 2.119 |
| REACT1ny Yes vs No | 1.048 | 0.502 | 2.186 |
| REACT2ny Yes vs No | >999.999 | <0.001 | >999.999 |
| REACT3ny Yes vs No | 0.515 | 0.132 | 2.011 |
| REACT4ny Yes vs No | 1.087 | 0.393 | 3.005 |
| REACT5ny Yes vs No | 0.425 | 0.026 | 6.938 |
| REACT6ny Yes vs No | 0.135 | 0.014 | 1.336 |
| INTENSMax Moderate vs Light | 0.527 | 0.169 | 1.640 |
| INTENSMax Severe vs Light | <0.001 | <0.001 | >999.999 |
| PQ3 Yes vs No | 0.893 | 0.390 | 2.044 |
| BETAPLUSny Yes vs No | 1.341 | 0.671 | 2.681 |

Supplementary Table 9. Logistic regression for persistence at 12 months (continuous independent variables)

| **Variable** | **Label** | **N** | **DF** | **Effect estimate** | **Standard error** | **Waldsches ChiSq** | **Pr > ChiSq** |
| --- | --- | --- | --- | --- | --- | --- | --- |
| age | Age (years) | 153 | 1 | 0.0469 | 0.0163 | 8.2518 | 0.0041 |
| bmi | BMI (kg/m²) | 138 | 1 | 0.0161 | 0.0323 | 0.2488 | 0.6179 |
| MEDHISTNumber | number of concomitant diseases | 153 | 1 | 0.0852 | 0.1145 | 0.5546 | 0.4565 |
| VITDDOSE0 | daily dosage of vitamin D intake at initial visit | 34 | 1 | 0.000024 | 0.000049 | 0.2446 | 0.6209 |
| timesince_diagnosis | Time since diagnosis (months) | 145 | 1 | 0.00774 | 0.00281 | 7.6139 | 0.0058 |
| Treatdur_CRF | Duration of treatment (days) | 152 | 1 | 0.000445 | 0.000146 | 9.2831 | 0.0023 |
| PQ4 | Injection site pain at initial visit (0:“No pain at all“ - 10: „Extremely severe pain) | 122 | 1 | -0.0969 | 0.0856 | 1.2823 | 0.2575 |
| SDMTNO0 | SDMT score at initial visit (0-110 correct answers) | 113 | 1 | 0.00677 | 0.0170 | 0.1585 | 0.6905 |
| sf36_3 | SF-36: limitations in physical activities because of health problems at initial visit (Standardized scale: 0-100) | 139 | 1 | -0.00990 | 0.00794 | 1.5551 | 0.2124 |
| sf36_4 | SF-36: limitations in usual role activities because of physical health problems at initial visit (Standardized scale: 0-100) | 139 | 1 | 0.00214 | 0.00966 | 0.0492 | 0.8245 |
| sf36_78 | SF-36: bodily pain at initial visit (Standardized scale: 0-100) | 125 | 1 | 0.00071 | 0.00955 | 0.0055 | 0.9408 |
| sf36_11 | SF-36: general health perceptions at initial visit (Standardized scale: 0-100) | 138 | 1 | 0.00995 | 0.0116 | 0.7327 | 0.3920 |
| sf36_9vit | SF-36: vitality at initial visit (Standardized scale: 0-100) | 140 | 1 | 0.00982 | 0.0104 | 0.8878 | 0.3461 |
| sf36_610 | SF-36: limitations in social activities because of physical or emotional problems at initial visit (Standardized scale: 0-100) | 139 | 1 | 0.0122 | 0.0247 | 0.2418 | 0.6229 |
| sf36_5 | SF-36: limitations in usual role activities because of emotional problems at initial visit (Standardized scale: 0-100) | 138 | 1 | 0.0106 | 0.00989 | 1.1379 | 0.2861 |
| sf36_9psy | SF-36: general mental health at initial visit (Standardized scale: 0-100) | 140 | 1 | 0.00976 | 0.0112 | 0.7601 | 0.3833 |
| cesd_sum | Depression (ADS-L) at initial visit (0:“No depressive symptoms“ - 60:“Maximum amount of depressive symptoms“) | 142 | 1 | -0.0103 | 0.0203 | 0.2568 | 0.6123 |
| weimus_ges | Fatigue at initial visit (total sum score from 0 to 68, with higher scores indicating higher degrees of fatigue) | 144 | 1 | -0.0160 | 0.0111 | 2.0628 | 0.1509 |
| weimus_kog | Cognitive fatigue at initial visit (subscore from 0 to 36) | 143 | 1 | -0.0297 | 0.0214 | 1.9163 | 0.1663 |
| weimus_koer | Physical fatigue at initial visit (subscore from 0 to 32) | 143 | 1 | -0.0274 | 0.0209 | 1.7208 | 0.1896 |
| msss | MSSS at initial visit (score from 14 to 84) | 145 | 1 | 0.00103 | 0.0158 | 0.0043 | 0.9479 |

The numbers of observations used (N) may vary from the total number of patients in the FAS, since patients with missing values for either the dependent and/or independent variables were not imputed and left out of the respective analysis.

# Detailed modelling results – persistence at 24 months

Supplementary Table 10. Logistic regression for persistence at 24 months (categorical independent variables)

| **Effect** | **Label** | **Reference** | **N** | **DF** | **Waldsches ChiSq** | **Pr > ChiSq** |
| --- | --- | --- | --- | --- | --- | --- |
| SEX | Sex (male, female) | 'Male' | 153 | 1 | 0.0524 | 0.8189 |
| MARITAL0 | Marital status (married/partnership, single) | 'Single' | 146 | 1 | 1.0390 | 0.3081 |
| EMPLOY0 | Employment status (employed, retired, keeping house, student, seeking work, self-employed, other, not reported) | 'Employed' | 145 | 7 | 5.3232 | 0.6206 |
| EDUCAT | Educational level (no certificate, elementary education, secondary education, university entrance qualification, college or university education, not reported) | 'Elementary education' | 142 | 5 | 1.7709 | 0.8799 |
| MEDHISTny | Concomitant diseases (no, yes) | 'No' | 153 | 1 | 0.8695 | 0.3511 |
| MEDHISTNumberga | Number of concomitant diseases (0,1,>=2) | '0' | 153 | 2 | 0.9361 | 0.6262 |
| EDSS0ga | Baseline EDSS (0-2.5,>=3) | '0 - 2.5' | 153 | 1 | 3.8964 | 0.0484 |
| EVENTNO0ga | Number of relapses during last two years prior to enrolment (missing/unknown,0,1,>1) | '0' | 153 | 3 | 6.9173 | 0.0746 |
| MSStatus | CIS vs. RRMS | 'CIS' | 153 | 1 | 0.2663 | 0.6058 |
| VITDny | Intake of vitamin D supplements at any visit (no, yes) | 'No' | 153 | 1 | 0.2574 | 0.6119 |
| VITOTHny | Intake of other nutrients or vitamins (no, yes) | 'No' | 153 | 1 | 0.6930 | 0.4051 |
| SMOKING | Smoking (never, past, present) | 'Never' | 142 | 2 | 1.3283 | 0.5147 |
| BETATREAT | New patients vs. patients already on Betaferon® | 'Betaferon-treatment-naïve' | 152 | 1 | 7.6105 | 0.0058 |
| ConcMedny | Concomitant medication (no, yes) | 'No' | 153 | 1 | 2.1280 | 0.1446 |
| CONCMEDnumberga | Number of concomitant medications (0,1,>=2) | '0' | 153 | 2 | 2.1291 | 0.3449 |
| BetacExpny | Previous usage of BETACONNECT™ (BETACONNECT-naïve, BETACONNECT-experienced) | 'BETACONNECT-naïve ' | 153 | 1 | 2.8641 | 0.0906 |
| PQ5 | Usage of electronic features of BETACONNECT (no, yes) | 'No' | 123 | 1 | 0.6105 | 0.4346 |
| SKINREACTny | Injection-site reactions (no, yes) | 'No' | 153 | 1 | 1.1951 | 0.2743 |
| REACT1ny | Injection-site reactions (redness; no,yes) | 'No' | 153 | 1 | 0.8385 | 0.3598 |
| REACT2ny | Injection-site reactions (other discoloration; no,yes) | 'No' | 153 | 1 | 0.0003 | 0.9870 |
| REACT3ny | Injection-site reactions (hematoma; no,yes) | 'No' | 153 | 1 | 0.0147 | 0.9036 |
| REACT4ny | Injection-site reactions (induration; no,yes) | 'No' | 153 | 1 | 0.6701 | 0.4130 |
| REACT5ny | Injection-site reactions (lipodystrophy; no,yes) | 'No' | 153 | 1 | 0.0106 | 0.9181 |
| REACT6ny | Injection-site reactions (necrosis; no,yes) | 'No' | 153 | 1 | 0.0006 | 0.9811 |
| INTENSMax | Worst intensity of local skin reactions at any visit (light, moderate, severe) | 'Light' | 61 | 2 | 1.1343 | 0.5671 |
| PQ3 | Flu-like symptoms (no, yes) | 'No' | 119 | 1 | 1.5536 | 0.2126 |
| BETAPLUSny | Participation in the Patient Support and Disease Management Program BETAPLUS® (no, yes) | 'No' | 153 | 1 | 0.0365 | 0.8485 |

The numbers of observations used (N) may vary from the total number of patients in the FAS, since patients with missing values for either the dependent and/or independent variables were not imputed and left out of the respective analysis.

Supplementary Table 11. Logistic regression for persistence at 24 months (categorical independent variables) – effect estimates

| **Effect** | **Odds ratio estimate** | **LowerCL** | **UpperCL** |
| --- | --- | --- | --- |
| SEX Female vs Male | 0.924 | 0.471 | 1.815 |
| MARITAL0 Married / with a partner vs Single | 0.665 | 0.303 | 1.457 |
| EMPLOY0 Keeping House vs Employed | 0.216 | 0.042 | 1.094 |
| EMPLOY0 Not reported vs Employed | 0.377 | 0.033 | 4.309 |
| EMPLOY0 Other vs Employed | <0.001 | <0.001 | >999.999 |
| EMPLOY0 Retired vs Employed | 0.981 | 0.391 | 2.464 |
| EMPLOY0 Seeking work vs Employed | 1.509 | 0.132 | 17.237 |
| EMPLOY0 Self-employed vs Employed | 1.887 | 0.348 | 10.230 |
| EMPLOY0 Student vs Employed | 0.503 | 0.080 | 3.154 |
| EDUCAT College or university education vs Elementary education | 1.067 | 0.346 | 3.284 |
| EDUCAT General certificate for secondary education vs Elementary education | 0.750 | 0.296 | 1.903 |
| EDUCAT No certificate vs Elementary education | <0.001 | <0.001 | >999.999 |
| EDUCAT Not reported vs Elementary education | 0.400 | 0.078 | 2.062 |
| EDUCAT University entrance qualification vs Elementary education | 0.778 | 0.201 | 3.008 |
| MEDHISTny Yes vs No | 1.355 | 0.716 | 2.565 |
| MEDHISTNumberga 1 vs 0 | 1.250 | 0.520 | 3.003 |
| MEDHISTNumberga >1 vs 0 | 1.421 | 0.683 | 2.956 |
| EDSS0ga 3 - 10 vs 0 - 2.5 | 2.371 | 1.006 | 5.586 |
| EVENTNO0ga 1 vs 0 | 0.388 | 0.171 | 0.881 |
| EVENTNO0ga >1 vs 0 | 0.334 | 0.113 | 0.989 |
| EVENTNO0ga Missing vs 0 | 0.591 | 0.251 | 1.390 |
| MSStatus RRMS vs CIS | 0.679 | 0.157 | 2.949 |
| VITDny Yes vs No | 1.180 | 0.622 | 2.239 |
| VITOTHny Yes vs No | 0.698 | 0.299 | 1.627 |
| SMOKING Current vs Never | 1.251 | 0.542 | 2.885 |
| SMOKING Former vs Never | 1.598 | 0.713 | 3.584 |
| BETATREAT already injecting Betaferon vs Betaferon-treatment-naïve | 2.949 | 1.368 | 6.357 |
| ConcMedny Yes vs No | 1.685 | 0.836 | 3.396 |
| CONCMEDnumberga 1 vs 0 | 1.667 | 0.643 | 4.317 |
| CONCMEDnumberga >1 vs 0 | 1.691 | 0.810 | 3.532 |
| BetacExpny Yes vs No | 1.767 | 0.914 | 3.417 |
| PQ5 Yes vs No | 0.649 | 0.220 | 1.919 |
| SKINREACTny Yes vs No | 1.440 | 0.749 | 2.769 |
| REACT1ny Yes vs No | 1.374 | 0.696 | 2.710 |
| REACT2ny Yes vs No | >999.999 | <0.001 | >999.999 |
| REACT3ny Yes vs No | 1.087 | 0.281 | 4.215 |
| REACT4ny Yes vs No | 1.484 | 0.577 | 3.816 |
| REACT5ny Yes vs No | 0.864 | 0.053 | 14.066 |
| REACT6ny Yes vs No | <0.001 | <0.001 | >999.999 |
| INTENSMax Moderate vs Light | 0.565 | 0.198 | 1.615 |
| INTENSMax Severe vs Light | <0.001 | <0.001 | >999.999 |
| PQ3 Yes vs No | 0.622 | 0.295 | 1.312 |
| BETAPLUSny Yes vs No | 1.064 | 0.563 | 2.011 |

Supplementary Table 12. Logistic regression for persistence at 24 months (continuous independent variables)

| **Variable** | **Label** | **N** | **DF** | **Effect estimate** | **Standard error** | **Waldsches ChiSq** | **Pr > ChiSq** |
| --- | --- | --- | --- | --- | --- | --- | --- |
| age | Age (years) | 153 | 1 | 0.0484 | 0.0149 | 10.4798 | 0.0012 |
| bmi | BMI (kg/m²) | 138 | 1 | -0.0197 | 0.0287 | 0.4747 | 0.4908 |
| MEDHISTNumber | number of concomitant diseases | 153 | 1 | 0.1020 | 0.1019 | 1.0011 | 0.3170 |
| VITDDOSE0 | daily dosage of vitamin D intake at initial visit | 34 | 1 | -0.00002 | 0.000028 | 0.4512 | 0.5018 |
| timesince_diagnosis | Time since diagnosis (months) | 145 | 1 | 0.00489 | 0.00212 | 5.3001 | 0.0213 |
| Treatdur_CRF | Duration of treatment (days) | 152 | 1 | 0.000245 | 0.000104 | 5.5755 | 0.0182 |
| PQ4 | Injection site pain at initial visit (0:“No pain at all“ - 10: „Extremely severe pain) | 122 | 1 | 0.00404 | 0.0776 | 0.0027 | 0.9584 |
| SDMTNO0 | SDMT score at initial visit (0-110 correct answers) | 113 | 1 | 0.0132 | 0.0159 | 0.6911 | 0.4058 |
| sf36_3 | SF-36: limitations in physical activities because of health problems at initial visit (Standardized scale: 0-100) | 139 | 1 | -0.00441 | 0.00646 | 0.4671 | 0.4943 |
| sf36_4 | SF-36: limitations in usual role activities because of physical health problems at initial visit (Standardized scale: 0-100) | 139 | 1 | 0.000157 | 0.00892 | 0.0003 | 0.9859 |
| sf36_78 | SF-36: bodily pain at initial visit (Standardized scale: 0-100) | 125 | 1 | -0.00458 | 0.00886 | 0.2674 | 0.6051 |
| sf36_11 | SF-36: general health perceptions at initial visit (Standardized scale: 0-100) | 138 | 1 | 0.00340 | 0.0106 | 0.1026 | 0.7488 |
| sf36_9vit | SF-36: vitality at initial visit (Standardized scale: 0-100) | 140 | 1 | 0.00946 | 0.00960 | 0.9696 | 0.3248 |
| sf36_610 | SF-36: limitations in social activities because of physical or emotional problems at initial visit (Standardized scale: 0-100) | 139 | 1 | 0.0167 | 0.0225 | 0.5507 | 0.4580 |
| sf36_5 | SF-36: limitations in usual role activities because of emotional problems at initial visit (Standardized scale: 0-100) | 138 | 1 | 0.0238 | 0.01000 | 5.6504 | 0.0175 |
| sf36_9psy | SF-36: general mental health at initial visit (Standardized scale: 0-100) | 140 | 1 | 0.0123 | 0.0105 | 1.3606 | 0.2434 |
| cesd_sum | Depression (ADS-L) at initial visit (0:“No depressive symptoms“ - 60:“Maximum amount of depressive symptoms“) | 142 | 1 | -0.00763 | 0.0187 | 0.1658 | 0.6838 |
| weimus_ges | Fatigue at initial visit (total sum score from 0 to 68, with higher scores indicating higher degrees of fatigue) | 144 | 1 | -0.00779 | 0.0103 | 0.5716 | 0.4496 |
| weimus_kog | Cognitive fatigue at initial visit (subscore from 0 to 36) | 143 | 1 | -0.0132 | 0.0201 | 0.4324 | 0.5108 |
| weimus_koer | Physical fatigue at initial visit (subscore from 0 to 32) | 143 | 1 | -0.0161 | 0.0193 | 0.6923 | 0.4054 |
| msss | MSSS at initial visit (score from 14 to 84) | 145 | 1 | 0.00934 | 0.0145 | 0.4135 | 0.5202 |

The numbers of observations used (N) may vary from the total number of patients in the FAS, since patients with missing values for either the dependent and/or independent variables were not imputed and left out of the respective analysis.

# Detailed modelling results – adherence at 12 months

Supplementary Table 13. Logistic regression for adherence at 12 months (categorical independent variables)

| **Effect** | **Label** | **Reference** | **N** | **DF** | **Waldsches ChiSq** | **Pr > ChiSq** |
| --- | --- | --- | --- | --- | --- | --- |
| SEX | Sex (male, female) | 'Male' | 119 | 1 | 5.3859 | 0.0203 |
| MARITAL0 | Marital status (married/partnership, single) | 'Single' | 114 | 1 | 0.0001 | 0.9932 |
| EMPLOY0 | Employment status (employed, retired, keeping house, student, seeking work, self-employed, other, not reported) | 'Employed' | 114 | 7 | 0.9127 | 0.9961 |
| EDUCAT | Educational level (no certificate, elementary education, secondary education, university entrance qualification, college or university education, not reported) | 'Elementary education' | 113 | 4 | 3.3624 | 0.4991 |
| MEDHISTny | Concomitant diseases (no, yes) | 'No' | 119 | 1 | 5.2924 | 0.0214 |
| MEDHISTNumberga | Number of concomitant diseases (0,1,>=2) | '0' | 119 | 2 | 5.4883 | 0.0643 |
| EDSS0ga | Baseline EDSS (0-2.5,>=3) | '0 - 2.5' | 119 | 1 | 0.2336 | 0.6289 |
| EVENTNO0ga | Number of relapses during last two years prior to enrolment (missing/unknown,0,1,>1) | '0' | 119 | 3 | 3.7612 | 0.2884 |
| MSStatus | CIS vs. RRMS | 'CIS' | 119 | 1 | 0.0284 | 0.8662 |
| VITDny | Intake of vitamin D supplements at any visit (no, yes) | 'No' | 119 | 1 | 1.6643 | 0.1970 |
| VITOTHny | Intake of other nutrients or vitamins (no, yes) | 'No' | 119 | 1 | 2.3088 | 0.1286 |
| SMOKING | Smoking (never, past, present) | 'Never' | 113 | 2 | 2.4971 | 0.2869 |
| BETATREAT | New patients vs. patients already on Betaferon® | 'Betaferon-treatment-naïve' | 119 | 1 | 1.7824 | 0.1819 |
| ConcMedny | Concomitant medication (no, yes) | 'No' | 119 | 1 | 0.0938 | 0.7594 |
| CONCMEDnumberga | Number of concomitant medications (0,1,>=2) | '0' | 119 | 2 | 0.4875 | 0.7837 |
| BetacExpny | Previous usage of BETACONNECT™ (BETACONNECT-naïve, BETACONNECT-experienced) | 'BETACONNECT-naïve ' | 119 | 1 | 0.7267 | 0.3940 |
| PQ5 | Usage of electronic features of BETACONNECT (no, yes) | 'No' | 98 | 1 | 4.3167 | 0.0377 |
| SKINREACTny | Injection-site reactions (no, yes) | 'No' | 119 | 1 | 0.1248 | 0.7239 |
| REACT1ny | Injection-site reactions (redness; no,yes) | 'No' | 119 | 1 | 0.0811 | 0.7758 |
| REACT2ny | Injection-site reactions (other discoloration; no,yes) | 'No' | 119 | 1 | 0.0005 | 0.9827 |
| REACT3ny | Injection-site reactions (hematoma; no,yes) | 'No' | 119 | 1 | 0.0284 | 0.8662 |
| REACT4ny | Injection-site reactions (induration; no,yes) | 'No' | 119 | 1 | 0.0089 | 0.9250 |
| REACT5ny | Injection-site reactions (lipodystrophy; no,yes) | 'No' | 119 | 1 | 0.3550 | 0.5513 |
| REACT6ny | Injection-site reactions (necrosis; no,yes) | 'No' | 119 | 1 | 0.0003 | 0.9871 |
| INTENSMax | Worst intensity of local skin reactions at any visit (light, moderate, severe) | 'Light' | 50 | 1 | 0.9699 | 0.3247 |
| PQ3 | Flu-like symptoms (no, yes) | 'No' | 96 | 1 | 0.4286 | 0.5127 |
| BETAPLUSny | Participation in the Patient Support and Disease Management Program BETAPLUS® (no, yes) | 'No' | 119 | 1 | 4.5013 | 0.0339 |

The numbers of observations used (N) may vary from the total number of patients in the FAS, since patients with missing values for either the dependent and/or independent variables were not imputed and left out of the respective analysis.

Supplementary Table 14. Logistic regression for adherence at 12 months (categorical independent variables) – effect estimates

| **Effect** | **Odds ratio estimate** | **LowerCL** | **UpperCL** |
| --- | --- | --- | --- |
| SEX Female vs Male | 2.642 | 1.163 | 6.000 |
| MARITAL0 Married / with a partner vs Single | 1.004 | 0.389 | 2.591 |
| EMPLOY0 Keeping House vs Employed | 0.962 | 0.083 | 11.114 |
| EMPLOY0 Not reported vs Employed | >999.999 | <0.001 | >999.999 |
| EMPLOY0 Other vs Employed | <0.001 | <0.001 | >999.999 |
| EMPLOY0 Retired vs Employed | 1.538 | 0.506 | 4.676 |
| EMPLOY0 Seeking work vs Employed | 0.481 | 0.029 | 8.006 |
| EMPLOY0 Self-employed vs Employed | >999.999 | <0.001 | >999.999 |
| EMPLOY0 Student vs Employed | 0.962 | 0.083 | 11.114 |
| EDUCAT College or university education vs Elementary education | 0.219 | 0.040 | 1.207 |
| EDUCAT General certificate for secondary education vs Elementary education | 0.250 | 0.052 | 1.202 |
| EDUCAT Not reported vs Elementary education | 0.250 | 0.026 | 2.362 |
| EDUCAT University entrance qualification vs Elementary education | 0.292 | 0.040 | 2.150 |
| MEDHISTny Yes vs No | 2.611 | 1.153 | 5.915 |
| MEDHISTNumberga 1 vs 0 | 3.333 | 1.013 | 10.971 |
| MEDHISTNumberga >1 vs 0 | 2.250 | 0.876 | 5.777 |
| EDSS0ga 3 - 10 vs 0 - 2.5 | 1.288 | 0.462 | 3.593 |
| EVENTNO0ga 1 vs 0 | 0.951 | 0.353 | 2.561 |
| EVENTNO0ga >1 vs 0 | 0.333 | 0.095 | 1.167 |
| EVENTNO0ga Missing vs 0 | 1.231 | 0.407 | 3.722 |
| MSStatus RRMS vs CIS | 1.162 | 0.203 | 6.648 |
| VITDny Yes vs No | 1.689 | 0.762 | 3.746 |
| VITOTHny Yes vs No | 0.483 | 0.189 | 1.235 |
| SMOKING Current vs Never | 2.100 | 0.680 | 6.485 |
| SMOKING Former vs Never | 1.823 | 0.697 | 4.766 |
| BETATREAT already injecting Betaferon vs Betaferon-treatment-naïve | 1.806 | 0.758 | 4.300 |
| ConcMedny Yes vs No | 0.870 | 0.355 | 2.127 |
| CONCMEDnumberga 1 vs 0 | 0.685 | 0.214 | 2.187 |
| CONCMEDnumberga >1 vs 0 | 0.947 | 0.371 | 2.420 |
| BetacExpny Yes vs No | 1.413 | 0.638 | 3.131 |
| PQ5 Yes vs No | 0.281 | 0.085 | 0.931 |
| SKINREACTny Yes vs No | 0.867 | 0.394 | 1.910 |
| REACT1ny Yes vs No | 0.889 | 0.397 | 1.991 |
| REACT2ny Yes vs No | >999.999 | <0.001 | >999.999 |
| REACT3ny Yes vs No | 0.861 | 0.150 | 4.925 |
| REACT4ny Yes vs No | 0.947 | 0.303 | 2.954 |
| REACT5ny Yes vs No | 0.427 | 0.026 | 7.020 |
| REACT6ny Yes vs No | <0.001 | <0.001 | >999.999 |
| INTENSMax Moderate vs Light | 0.545 | 0.163 | 1.822 |
| PQ3 Yes vs No | 1.366 | 0.537 | 3.473 |
| BETAPLUSny Yes vs No | 2.381 | 1.068 | 5.306 |

Supplementary Table 15. Logistic regression for adherence at 12 months (continuous independent variables)

| **Variable** | **Label** | **N** | **DF** | **Effect estimate** | **Standard error** | **Waldsches ChiSq** | **Pr > ChiSq** |
| --- | --- | --- | --- | --- | --- | --- | --- |
| age | Age (years) | 119 | 1 | 0.0192 | 0.0172 | 1.2507 | 0.2634 |
| bmi | BMI (kg/m²) | 110 | 1 | 0.00786 | 0.0395 | 0.0395 | 0.8424 |
| MEDHISTNumber | number of concomitant diseases | 119 | 1 | 0.3429 | 0.1716 | 3.9913 | 0.0457 |
| VITDDOSE0 | daily dosage of vitamin D intake at initial visit | 29 | 1 | 0.000019 | 0.000042 | 0.1969 | 0.6573 |
| timesince_diagnosis | Time since diagnosis (months) | 113 | 1 | 0.00122 | 0.00246 | 0.2453 | 0.6204 |
| Treatdur_CRF | Duration of treatment (days) | 118 | 1 | 0.000201 | 0.000132 | 2.3071 | 0.1288 |
| PQ4 | Injection site pain at initial visit (0:“No pain at all“ - 10: „Extremely severe pain) | 98 | 1 | -0.0588 | 0.1028 | 0.3279 | 0.5669 |
| SDMTNO0 | SDMT score at initial visit (0-110 correct answers) | 86 | 1 | -0.00981 | 0.0216 | 0.2071 | 0.6491 |
| sf36_3 | SF-36: limitations in physical activities because of health problems at initial visit (Standardized scale: 0-100) | 112 | 1 | -0.00748 | 0.00821 | 0.8286 | 0.3627 |
| sf36_4 | SF-36: limitations in usual role activities because of physical health problems at initial visit (Standardized scale: 0-100) | 112 | 1 | 0.00475 | 0.0103 | 0.2130 | 0.6444 |
| sf36_78 | SF-36: bodily pain at initial visit (Standardized scale: 0-100) | 102 | 1 | -0.00437 | 0.0107 | 0.1663 | 0.6834 |
| sf36_11 | SF-36: general health perceptions at initial visit (Standardized scale: 0-100) | 111 | 1 | -0.00163 | 0.0131 | 0.0155 | 0.9008 |
| sf36_9vit | SF-36: vitality at initial visit (Standardized scale: 0-100) | 113 | 1 | -0.00508 | 0.0114 | 0.1974 | 0.6568 |
| sf36_610 | SF-36: limitations in social activities because of physical or emotional problems at initial visit (Standardized scale: 0-100) | 112 | 1 | -0.0177 | 0.0269 | 0.4341 | 0.5100 |
| sf36_5 | SF-36: limitations in usual role activities because of emotional problems at initial visit (Standardized scale: 0-100) | 111 | 1 | 0.000191 | 0.0113 | 0.0003 | 0.9865 |
| sf36_9psy | SF-36: general mental health at initial visit (Standardized scale: 0-100) | 113 | 1 | -0.0167 | 0.0134 | 1.5549 | 0.2124 |
| cesd_sum | Depression (ADS-L) at initial visit (0:“No depressive symptoms“ - 60:“Maximum amount of depressive symptoms“) | 114 | 1 | 0.0336 | 0.0254 | 1.7508 | 0.1858 |
| weimus_ges | Fatigue at initial visit (total sum score from 0 to 68, with higher scores indicating higher degrees of fatigue) | 117 | 1 | -0.00921 | 0.0119 | 0.5975 | 0.4395 |
| weimus_kog | Cognitive fatigue at initial visit (subscore from 0 to 36) | 116 | 1 | -0.0148 | 0.0228 | 0.4237 | 0.5151 |
| weimus_koer | Physical fatigue at initial visit (subscore from 0 to 32) | 116 | 1 | -0.0173 | 0.0225 | 0.5921 | 0.4416 |
| msss | MSSS at initial visit (score from 14 to 84) | 117 | 1 | -0.00942 | 0.0177 | 0.2846 | 0.5937 |

The numbers of observations used (N) may vary from the total number of patients in the FAS, since patients with missing values for either the dependent and/or independent variables were not imputed and left out of the respective analysis.

# Detailed modelling results – adherence at 24 months

Supplementary Table 16. Logistic regression for adherence at 24 months (categorical independent variables)

| **Effect** | **Label** | **Reference** | **N** | **DF** | **Waldsches ChiSq** | **Pr > ChiSq** |
| --- | --- | --- | --- | --- | --- | --- |
| SEX | Sex (male, female) | 'Male' | 120 | 1 | 0.0047 | 0.9456 |
| MARITAL0 | Marital status (married/partnership, single) | 'Single' | 115 | 1 | 0.2451 | 0.6205 |
| EMPLOY0 | Employment status (employed, retired, keeping house, student, seeking work, self-employed, other, not reported) | 'Employed' | 115 | 7 | 3.2435 | 0.8616 |
| EDUCAT | Educational level (no certificate, elementary education, secondary education, university entrance qualification, college or university education, not reported) | 'Elementary education' | 114 | 4 | 3.6059 | 0.4620 |
| MEDHISTny | Concomitant diseases (no, yes) | 'No' | 120 | 1 | 0.5284 | 0.4673 |
| MEDHISTNumberga | Number of concomitant diseases (0,1,>=2) | '0' | 120 | 2 | 0.6322 | 0.7290 |
| EDSS0ga | Baseline EDSS (0-2.5,>=3) | '0 - 2.5' | 120 | 1 | 2.0912 | 0.1482 |
| EVENTNO0ga | Number of relapses during last two years prior to enrolment (missing/unknown,0,1,>1) | '0' | 120 | 3 | 4.8060 | 0.1866 |
| MSStatus | CIS vs. RRMS | 'CIS' | 120 | 1 | 0.0018 | 0.9666 |
| VITDny | Intake of vitamin D supplements at any visit (no, yes) | 'No' | 120 | 1 | 0.0356 | 0.8503 |
| VITOTHny | Intake of other nutrients or vitamins (no, yes) | 'No' | 120 | 1 | 0.3671 | 0.5446 |
| SMOKING | Smoking (never, past, present) | 'Never' | 114 | 2 | 4.3154 | 0.1156 |
| BETATREAT | New patients vs. patients already on Betaferon® | 'Betaferon-treatment-naïve' | 120 | 1 | 7.5940 | 0.0059 |
| ConcMedny | Concomitant medication (no, yes) | 'No' | 120 | 1 | 0.8240 | 0.3640 |
| CONCMEDnumberga | Number of concomitant medications (0,1,>=2) | '0' | 120 | 2 | 1.2854 | 0.5259 |
| BetacExpny | Previous usage of BETACONNECT™ (BETACONNECT-naïve, BETACONNECT-experienced) | 'BETACONNECT-naïve ' | 120 | 1 | 4.3786 | 0.0364 |
| PQ5 | Usage of electronic features of BETACONNECT (no, yes) | 'No' | 98 | 1 | 2.8046 | 0.0940 |
| SKINREACTny | Injection-site reactions (no, yes) | 'No' | 120 | 1 | 1.1632 | 0.2808 |
| REACT1ny | Injection-site reactions (redness; no,yes) | 'No' | 120 | 1 | 0.8019 | 0.3705 |
| REACT2ny | Injection-site reactions (other discoloration; no,yes) | 'No' | 120 | 1 | 0.0003 | 0.9867 |
| REACT3ny | Injection-site reactions (hematoma; no,yes) | 'No' | 120 | 1 | 0.7420 | 0.3890 |
| REACT4ny | Injection-site reactions (induration; no,yes) | 'No' | 120 | 1 | 0.7309 | 0.3926 |
| REACT5ny | Injection-site reactions (lipodystrophy; no,yes) | 'No' | 120 | 1 | 0.0006 | 0.9810 |
| REACT6ny | Injection-site reactions (necrosis; no,yes) | 'No' | 120 | 1 | 0.0001 | 0.9907 |
| INTENSMax | Worst intensity of local skin reactions at any visit (light, moderate, severe) | 'Light' | 51 | 1 | 0.0001 | 0.9910 |
| PQ3 | Flu-like symptoms (no, yes) | 'No' | 97 | 1 | 0.0598 | 0.8068 |
| BETAPLUSny | Participation in the Patient Support and Disease Management Program BETAPLUS® (no, yes) | 'No' | 120 | 1 | 2.1799 | 0.1398 |

Supplementary Table 17. Logistic regression for adherence at 24 months (categorical independent variables) – effect estimates

| **Effect** | **Odds ratio estimate** | **LowerCL** | **UpperCL** |
| --- | --- | --- | --- |
| SEX Female vs Male | 1.027 | 0.478 | 2.205 |
| MARITAL0 Married / with a partner vs Single | 0.801 | 0.334 | 1.925 |
| EMPLOY0 Keeping House vs Employed | 0.569 | 0.050 | 6.545 |
| EMPLOY0 Not reported vs Employed | 1.139 | 0.069 | 18.874 |
| EMPLOY0 Other vs Employed | <0.001 | <0.001 | >999.999 |
| EMPLOY0 Retired vs Employed | 1.519 | 0.574 | 4.019 |
| EMPLOY0 Seeking work vs Employed | 2.278 | 0.198 | 26.181 |
| EMPLOY0 Self-employed vs Employed | 4.556 | 0.487 | 42.645 |
| EMPLOY0 Student vs Employed | 0.569 | 0.050 | 6.545 |
| EDUCAT College or university education vs Elementary education | 0.486 | 0.139 | 1.704 |
| EDUCAT General certificate for secondary education vs Elementary education | 0.604 | 0.208 | 1.756 |
| EDUCAT Not reported vs Elementary education | 0.292 | 0.042 | 2.023 |
| EDUCAT University entrance qualification vs Elementary education | 0.250 | 0.048 | 1.293 |
| MEDHISTny Yes vs No | 1.305 | 0.637 | 2.674 |
| MEDHISTNumberga 1 vs 0 | 1.179 | 0.458 | 3.034 |
| MEDHISTNumberga >1 vs 0 | 1.400 | 0.608 | 3.223 |
| EDSS0ga 3 - 10 vs 0 - 2.5 | 1.970 | 0.786 | 4.936 |
| EVENTNO0ga 1 vs 0 | 0.385 | 0.153 | 0.969 |
| EVENTNO0ga >1 vs 0 | 0.438 | 0.126 | 1.525 |
| EVENTNO0ga Missing vs 0 | 0.758 | 0.290 | 1.986 |
| MSStatus RRMS vs CIS | 0.966 | 0.187 | 4.987 |
| VITDny Yes vs No | 0.933 | 0.456 | 1.910 |
| VITOTHny Yes vs No | 0.754 | 0.302 | 1.882 |
| SMOKING Current vs Never | 2.464 | 0.923 | 6.573 |
| SMOKING Former vs Never | 2.006 | 0.841 | 4.788 |
| BETATREAT already injecting Betaferon vs Betaferon-treatment-naïve | 3.596 | 1.447 | 8.937 |
| ConcMedny Yes vs No | 1.454 | 0.648 | 3.263 |
| CONCMEDnumberga 1 vs 0 | 1.131 | 0.382 | 3.353 |
| CONCMEDnumberga >1 vs 0 | 1.583 | 0.680 | 3.687 |
| BetacExpny Yes vs No | 2.239 | 1.052 | 4.763 |
| PQ5 Yes vs No | 0.343 | 0.098 | 1.200 |
| SKINREACTny Yes vs No | 1.492 | 0.721 | 3.089 |
| REACT1ny Yes vs No | 1.406 | 0.667 | 2.962 |
| REACT2ny Yes vs No | >999.999 | <0.001 | >999.999 |
| REACT3ny Yes vs No | 2.145 | 0.378 | 12.181 |
| REACT4ny Yes vs No | 1.574 | 0.556 | 4.456 |
| REACT5ny Yes vs No | 1.034 | 0.063 | 16.930 |
| REACT6ny Yes vs No | <0.001 | <0.001 | >999.999 |
| INTENSMax Moderate vs Light | 1.007 | 0.325 | 3.115 |
| PQ3 Yes vs No | 0.903 | 0.399 | 2.042 |
| BETAPLUSny Yes vs No | 1.727 | 0.836 | 3.568 |

Note on “>999.999”: There are not enough meaningful cases for these characteristics

Supplementary Table 18. Logistic regression for adherence at 24 months (continuous independent variables)

| **Variable** | **Label** | **N** | **DF** | **Effect estimate** | **Standarderror** | **Waldsches ChiSq** | **Pr > ChiSq** |
| --- | --- | --- | --- | --- | --- | --- | --- |
| age | Age (years) | 120 | 1 | 0.0326 | 0.0161 | 4.1358 | 0.0420 |
| bmi | BMI (kg/m²) | 111 | 1 | -0.00464 | 0.0355 | 0.0170 | 0.8962 |
| MEDHISTNumber | number of concomitant diseases | 120 | 1 | 0.1954 | 0.1238 | 2.4889 | 0.1147 |
| VITDDOSE0 | daily dosage of vitamin D intake at initial visit | 29 | 1 | -0.00001 | 0.000020 | 0.4915 | 0.4832 |
| timesince_diagnosis | Time since diagnosis (months) | 114 | 1 | 0.00209 | 0.00222 | 0.8906 | 0.3453 |
| Treatdur_CRF | Duration of treatment (days) | 119 | 1 | 0.000161 | 0.000110 | 2.1379 | 0.1437 |
| PQ4 | Injection site pain at initial visit (0:“No pain at all“ - 10: „Extremely severe pain) | 99 | 1 | 0.00910 | 0.0897 | 0.0103 | 0.9192 |
| SDMTNO0 | SDMT score at initial visit (0-110 correct answers) | 87 | 1 | -0.00681 | 0.0193 | 0.1239 | 0.7249 |
| sf36_3 | SF-36: limitations in physical activities because of health problems at initial visit (Standardized scale: 0-100) | 113 | 1 | -0.00352 | 0.00691 | 0.2589 | 0.6109 |
| sf36_4 | SF-36: limitations in usual role activities because of physical health problems at initial visit (Standardized scale: 0-100) | 113 | 1 | 0.00570 | 0.00965 | 0.3486 | 0.5549 |
| sf36_78 | SF-36: bodily pain at initial visit (Standardized scale: 0-100) | 103 | 1 | -0.0102 | 0.00991 | 1.0494 | 0.3056 |
| sf36_11 | SF-36: general health perceptions at initial visit (Standardized scale: 0-100) | 112 | 1 | -0.00821 | 0.0121 | 0.4609 | 0.4972 |
| sf36_9vit | SF-36: vitality at initial visit (Standardized scale: 0-100) | 114 | 1 | 0.000794 | 0.0104 | 0.0058 | 0.9391 |
| sf36_610 | SF-36: limitations in social activities because of physical or emotional problems at initial visit (Standardized scale: 0-100) | 113 | 1 | 0.00815 | 0.0236 | 0.1197 | 0.7294 |
| sf36_5 | SF-36: limitations in usual role activities because of emotional problems at initial visit (Standardized scale: 0-100) | 112 | 1 | 0.0194 | 0.0109 | 3.1492 | 0.0760 |
| sf36_9psy | SF-36: general mental health at initial visit (Standardized scale: 0-100) | 114 | 1 | -0.00375 | 0.0113 | 0.1095 | 0.7407 |
| cesd_sum | Depression (ADS-L) at initial visit (0:“No depressive symptoms“ - 60:“Maximum amount of depressive symptoms“) | 115 | 1 | 0.0200 | 0.0213 | 0.8856 | 0.3467 |
| weimus_ges | Fatigue at initial visit (total sum score from 0 to 68, with higher scores indicating higher degrees of fatigue) | 118 | 1 | 0.000581 | 0.0111 | 0.0027 | 0.9582 |
| weimus_kog | Cognitive fatigue at initial visit (subscore from 0 to 36) | 117 | 1 | 0.00502 | 0.0214 | 0.0551 | 0.8145 |
| weimus_koer | Physical fatigue at initial visit (subscore from 0 to 32) | 117 | 1 | -0.00395 | 0.0210 | 0.0353 | 0.8510 |
| msss | MSSS at initial visit (score from 14 to 84) | 118 | 1 | 0.00905 | 0.0159 | 0.3243 | 0.5690 |

The numbers of observations used (N) may vary from the total number of patients in the FAS, since patients with missing values for either the dependent and/or independent variables were not imputed and left out of the respective analysis.
